# Epitope-Based Nipah Virus G and F Head-to-Head Dimer mRNA Vaccines Exhibit Distinct Immunogenicity and Immune Profiles

**DOI:** 10.3390/vaccines14080718

**Published:** 2026-08-20

**Authors:** Rui Peng, Jiali Xu, Longhai Yuan, Bai Li, Hongyu Chen, Cong Tang, Hao Yang, Yanan Zhou, Yun Yang, Qing Huang, Junbin Wang, Shuaiyao Lu

**Affiliations:** 1 Institute of Medical Biology, Chinese Academy of Medical Sciences & Peking Union Medical College, Kunming 650118, China; s2024018032@student.pumc.edu.cn (R.P.); 12024214162@stu.ynu.edu.cn (J.X.); s2023018032@pumc.edu.cn (L.Y.); libai2021@imbcams.com.cn (B.L.); chenhy@imbcams.com.cn (H.C.); tangc_213@imbcams.com.cn (C.T.); yanghao0222@imbcams.com.cn (H.Y.); 15887034059@163.com (Y.Z.); yangyunimbcams@163.com (Y.Y.); qinghuangfgh@163.com (Q.H.); 2 Yunnan Key Laboratory of Cross-Border Infectious Disease Control and Prevention and Novel Drug Development, Yunnan University, Kunming 650118, China; 3 State Key Laboratory of Respiratory Health and Multimorbidity, Beijing 100005, China; 4 Key Laboratory of Pathogen Infection Prevention and Control, Ministry of Education, Peking Union Medical College, Beijing 100730, China; 5 Yunnan Provincial Key Laboratory of Vector-Borne Diseases Control and Research, Kunming 650118, China

**Keywords:** Nipah virus, paramyxovirus, mRNA vaccine, antigen design

## Abstract

**Background/Objectives:** Nipah virus (NiV) is a highly pathogenic zoonotic agent that causes fatal respiratory and neurological diseases, for which no approved vaccines are currently available. To address this unmet need, we developed and evaluated novel mRNA-LNP vaccine candidates. These vaccines employ an epitope- and structure–guided “immune-focusing” strategy, aiming to maximize the exposure of critical neutralizing epitopes on the viral attachment (G) and fusion (F) glycoproteins. **Methods:** Head-to-head dimeric antigens (2Gs and 2Fs) were engineered by removing subdominant stalk regions and tandemly duplicating epitope-rich globular head domains. Three LNP-encapsulated candidates-NV1 (encoding 2Gs), NV2 (encoding 2Fs), and NV3 (a 1:1 mixture of NV1 and NV2)-were evaluated in BALB/c mice and Syrian hamsters to systematically assess humoral responses, cytokine secretion, BCR/TCR repertoires, and systemic safety. **Results:** NV1 and NV3 induced potent binding antibodies and strong neutralizing activity against NiV pseudoviruses. Meanwhile, although NV2 lacked neutralizing activity, it induced superior Th1-biased cellular immunity, accompanied by extensive T-cell clonal proliferation. BCR/TCR sequencing revealed unique adaptive immune characteristics that perfectly align with the distinct immunogenic properties described above. Preliminary safety assessments found no toxicity to vital organs, while active germinal center formation confirmed the success of immune mobilization. **Conclusions:** An epitope-centered dimer design effectively shapes the unique defense mechanisms of the adaptive immune system. NV3 offers a balanced and synergistic strategy that combines potent humoral and cellular immune defenses, providing a highly promising platform for the development of a Nipah virus (NiV) vaccine.

## 1. Introduction

Nipah virus (NiV) is a highly pathogenic zoonotic virus belonging to the genus *Henipavirus* within the family *Paramyxoviridae* [1]. It first emerged in Malaysia in 1998, causing an outbreak that resulted in over 100 deaths [2]. Since then, NiV has caused periodic outbreaks in Southeast and South Asia, particularly in Bangladesh and India [3,4]. Genomic epidemiology distinguishes two main lineages: the Malaysia (NiV-M) and Bangladesh (NiV-B) clades [1,5,6]. According to the World Health Organization (WHO) surveillance, global NiV outbreaks remain primarily endemic to India and Bangladesh. In 2025, India reported a 50% CFR among four confirmed cases, while Bangladesh observed a 100% CFR (case fatality rate) for its four reported cases. The cumulative burden in Bangladesh has reached 347 cases, with the CFR stabilizing at a significant 71.7% [7]. On 26 January 2026, India notified the WHO of two laboratory-confirmed cases of NiV infection in West Bengal [8]; on 3 February 2026 (reported on 6 February), Bangladesh confirmed one additional fatal case [9]. Thus, between 2025 and early 2026, India and Bangladesh reported a total of 11 confirmed cases, including 8 fatalities. Driven by its substantial fatality rate, potential for cross-species spillover, and risk of person-to-person spread, the WHO has classified NiV as a top-priority pathogen demanding expedited research and development [10].

Although *Pteropus* fruit bats serve as the primary ecological reservoir of NiV, viral spillover to human populations is mediated by intermediate hosts (e.g., swine), ingestion of tainted foodstuffs (such as raw date palm sap), or direct person-to-person spread [11]. The clinical presentation of NiV infection is complex and highly severe. Following an incubation period of 4–14 days, patients typically present with non-specific influenza-like symptoms, including fever, headache, myalgia, and vomiting, which significantly complicates early clinical diagnosis. The disease progresses rapidly, exhibiting broad tissue tropism. Beyond causing acute respiratory distress syndrome (ARDS), the virus displays strong neurotropism leading to acute encephalitis, characterized by altered consciousness, seizures, and coma. Even among survivors, approximately 20% suffer from long-term neurological sequelae, such as brain atrophy and facial paralysis [12,13], with some cases developing relapsing encephalitis months or years later. This high mortality and morbidity make NiV one of the most severe challenges to global public health.

Possessing an unsegmented, negative-strand, single-stranded RNA genome of roughly 18.2 kb, NiV directs the expression of six structural proteins arranged linearly from 3′ to 5′: nucleocapsid (N), phosphoprotein (P), matrix protein (M), fusion protein (F), attachment glycoprotein (G), and large polymerase (L) [14]. Its entry relies on the synergistic action of the G and F proteins. The surface glycoprotein specifically recognizes and binds to highly conserved host cellular receptors-Ephrin-B2 and Ephrin-B3. This binding triggers a conformational transition in the G protein, which subsequently induces an irreversible structural rearrangement in the F protein, mediating fusion between the viral envelope and the host cell membrane [15,16]. Consequently, the G and F proteins are recognized as critical antigen targets for vaccine development. Studies indicate that the G protein primarily elicits high levels of neutralizing antibodies, while the stabilized F protein significantly enhances the breadth of immune protection [17]. Previous research leveraged the ChAdOx1 vector to encode the NiV-B G protein, resulting in a viral-vectored vaccine that elicited high-titer neutralizing antibodies in Syrian hamsters. This vaccine conferred complete protection against lethal NiV-M challenge, further consolidating the finding that the G protein is highly potent in inducing robust neutralizing antibody responses [18]. Current R&D strategies are increasingly shifting from single-antigen approaches to combined F/G designs or structural optimization to overcome the limitations of limited immunogenicity and non-durable protection associated with single antigens [17,19,20].

In light of the urgent threat posed by NiV, the mRNA vaccine platform demonstrates significant potential. The successful deployment of mRNA technology during the COVID-19 pandemic demonstrated its rapid response capabilities and potent immune activation [21,22]. Significant progress has also been made in developing mRNA vaccines for various pathogens, including influenza virus [23], mpox virus [24], and respiratory syncytial virus (RSV) [25,26]. This platform utilizes delivery systems, such as lipid nanoparticles (LNPs), to transport mRNA-encoding specific antigens into the host body. Host cells translate the mRNA into antigenic proteins, which are then processed via endosomal pathways to effectively activate T cells and B cells, thereby inducing robust and balanced humoral and cellular immune responses [27,28].

In this study, we employed an epitope-based and structure-guided antigen design strategy to systematically analyze dominant immunogenic epitopes of the NiV F and G proteins. To maximize the exposure of key neutralizing epitopes, we designed novel head-to-head dimer antigens, designated 2F and 2G, by tandemly repeating the epitope-rich head domains of the NiV F and G proteins, respectively, without any intervening linkers. Based on this design, we developed three candidate mRNA vaccines: NV1 (encoding 2Gs), NV2 (encoding 2Fs), and NV3 (a 1:1 mixture of NV1 vaccine and NV2 vaccine). Immunogenicity assessments in BALB/c mice and Syrian hamsters revealed that NV1 favored the induction of strong humoral immune responses, while NV2 was superior in eliciting cellular immunity. NV3 exhibited balanced immunogenicity, capable of stimulating potent humoral and cellular responses simultaneously.

To further investigate the impact of different antigen designs on the immune repertoire, we performed deep sequencing of T-cell receptor (TCR) and B-cell receptor (BCR) repertoires. The results indicated that the three vaccine candidates reshaped the immune repertoire with distinct distribution characteristics, providing a molecular-level explanation for the observed differences in immunogenicity. Furthermore, preliminary safety evaluations revealed no vaccine-related toxicity or multi-organ damage. In conclusion, by utilizing an epitope-based and structure-guided design strategy, we successfully developed three mRNA vaccine candidates with favorable immunogenicity. We provide a preliminary analysis of their immune profiles and safety characteristics, offering new perspectives and strategies for NiV vaccine design.

## 2. Materials and Methods

### 2.1. Predicted Structures of Antigens and Characterization of Immunogenic Epitopes

The structures of antigens 2Gs pTM=0.57 and 2Fs pTM=0.55, as well as the G pTM=0.71 and F pTM=0.7 proteins, were predicted using the AlphaFold3 web server (https://alphafoldserver.com (accessed on 19 June 2025)) [29]. Linear B-cell epitopes and MHC class I/II epitopes for each protein were predicted using the IEDB resource [30]. All prediction results were visualized in PyMOL 3.0.3 software. Antigen-antibody complexes (G docked to antibody from PDB 8CX4; F docked to antibody from PDB 6TYS) were modeled primarily using the AlphaFold3 web server. Specifically, the heavy- and light-chain variable region sequences of the antibodies were retrieved from the Protein Data Bank (PDB IDs: 8CX4 and 6TYS). The amino acid sequences of the F/G antigens and the corresponding antibody heavy- and light-chain sequences were then submitted to the AlphaFold3 multimer prediction function. Following prediction, the highest-confidence model was selected and visualized/analyzed in PyMOL.

### 2.2. Animals and Ethics Statement

Female BALB/c mice and Syrian hamsters (6–8 weeks of age) were obtained from the Institute of Medical Biology, Chinese Academy of Medical Sciences, and Beijing Vital River Laboratory Animal Technology Co., Ltd. (Beijing, China). (License: SCXK (Jing) 2021-0011), respectively. All animals were housed in a barrier facility under standard specific pathogen-free (SPF) conditions. All in vivo procedures strictly complied with the NIH Guidelines for Laboratory Animal Welfare (NIH Publication No. 8023) and were officially approved by the Institutional Animal Care and Use Committee (IACUC) of the Institute of Medical Biology, CAMS (Permit numbers: DWSP202207005 for mice, approved 11 July 2022; DWSP202603055 for hamsters, approved 23 March 2026). Following a 1-week acclimatization period, both mice and Syrian hamsters were randomly allocated into 7 experimental groups per species (*n* = 5 per group) via a simple random assignment sequence. This randomization was conducted to equalize baseline parameters (such as body weight) across groups and minimize potential confounders prior to immunization. To minimize experimental bias, blinding was implemented during animal handling, sample collection, and subsequent immunological and pathological analyses.

### 2.3. Cells

HEK 293T (#CL-0005) and Vero (#CL-0242) cell lines were acquired from Procell Life Science & Technology Co., Ltd. (Wuhan, China), and cryobanked at our facility. For in vitro experiments, both lines were cultivated in Dulbecco’s modified Eagle’s medium (DMEM, Gibco, Grand Island, NY, USA, #8123187) containing 10% heat-inactivated fetal bovine serum (FBS, Gibco, Grand Island, NY, USA, #2440086) alongside a 1% penicillin-streptomycin solution, and kept in a standard 37 °C environment with 5% CO_2_.

### 2.4. Generation of NiV Pseudotyped Viruses

Nipah pseudotyped viruses (NiV-M strain) were generated using a vesicular stomatitis virus (VSV) backbone system. Briefly, HEK 293T cells were seeded in a T75 flask one day prior to transfection to achieve approximately 90% confluence. For transfection, 75 µL of LipoFilter transfection reagent was diluted in 0.75 mL of Opti-MEM (Tube A) and incubated for 5 min at room temperature. Concurrently, 10 µg of NiV-M F plasmid and 20 µg of NiV-M G plasmid were diluted in 0.75 mL of Opti-MEM (Tube B) and incubated for 5 min. Tubes A and B were then mixed gently and incubated for 15–20 min to allow for complex formation. Meanwhile, the original culture medium of the HEK 293T cells was discarded and replaced with fresh medium containing the ΔG-VSV backbone pseudovirus, after which the transfection mixture was added along the side wall of the flask and mixed gently. After incubation at 37 °C in a 5% CO_2_ humidified incubator for 6–8 h, the culture supernatant was replaced with fresh DMEM and incubated for an additional 40–48 h. Once cell rounding, fragmentation, and culture medium acidification (yellowing) were observed, the supernatant was harvested, centrifuged at 4000 rpm for 10 min, and filtered through a 0.22 µm membrane filter. The resulting pseudoviruses were aliquoted and stored at −80 °C until further use.

### 2.5. Design, Preparation and Characterization of mRNA-LNPs

The optimized DNA sequence encoding 2Gs/2Fs, flanked by 5′ and 3′ untranslated regions (UTRs) and a poly(A) tail, was cloned into a plasmid under the control of a T7 promoter and linearized to serve as the template for in vitro transcription (IVT). To optimize target antigen expression, the specific 5′ and 3′ UTRs incorporated were derived from the Pfizer-BioNTech SARS-CoV-2 vaccine (BNT162b2). Furthermore, a sequence encoding the mouse IL-6 signal peptide (AUGAAAUUUCUGAGCGCUAGGGACUUUCACCCAGUGGCUUUCCUGGGCCUGAUGCUGGUGACCACCACCGCC) was fused upstream of the 2Gs/2Fs construct to direct protein secretion. The mRNA was synthesized via IVT using the EasyCap T7 Co-transcription Kit with CAG Trimer (Vazyme, Nanjing, China, #DD4203-00), which utilizes T7 RNA polymerase. During the transcription reaction, standard UTP was fully substituted with N1-methylpseudouridine-5′-triphosphate (N1-methylpseudo-UTP, Novoprotein). Co-transcriptional capping of the mRNA (Cap 1 structure) was achieved in a single step using the CAG trimer cap analog included in the kit. The complete mRNA sequence details are provided in the Supplementary Information (Figures S1 and S2).

The purified mRNA was dissolved in 50 mM sodium acetate buffer (pH 4.0) to a final concentration of 100–120 ng/uL (aqueous phase). To prepare the lipid phase, SM-102, DSPC, cholesterol, and DMG-PEG 2000 were solubilized in anhydrous ethanol at a specified molar ratio of 50:10:38.5:10, attaining a final total lipid concentration of 16 mM (organic phase). mRNA-LNPs were formulated by microfluidic mixing of the aqueous and organic phases at a volume ratio of 3:1. The resulting mixture was subjected to ultrafiltration using a 100 kDa molecular weight cut-off membrane to remove residual ethanol and exchange the buffer into 20 mM Tris-HCl. Following encapsulation, the mRNA-LNPs were diluted 20-fold for particle size analysis using a Malvern Zetasizer (Malvern, United Kingdom). The encapsulation efficiency (EE) was quantified with the Quant-iT^TM^ RiboGreen^TM^ RNA Reagent (Thermo Fisher Scientific, Waltham, MA, USA, #R11490). The stability of the mRNA-LNPs at 4 °C was preliminarily assessed by monitoring changes in size and EE at specific time intervals (6 h, 24 h, 48 h, 72 h, and 96 h post-initial immunization) after preparation. Morphological characterization was performed by transmission electron microscopy (TEM) using a Talos F200C microscope (Thermo Fisher Scientific, Waltham, MA, USA) equipped with a Ceta 4 K × 4 K camera, with technical support from Scientific Compass Service Platform. All experimental LNPs were freshly synthesized prior to immunization and cellular assays.

### 2.6. Cell Transfection and Western Blot Analysis

For cell transfection, HEK 293T cells were treated with either mRNA using a commercial mRNA liposome transfection reagent (Thermo Fisher Scientific, Waltham, MA, USA, #L3000015) (2.5 µg of mRNA per well) or directly incubated with mRNA-LNPs (5 µg of mRNA per well), designated as “mRNA” and “mRNA-LNP”, respectively. After 24 h, cells were harvested and lysed to obtain total protein lysates. To promote full denaturation, protein specimens were diluted in SDS sample loading buffer and heat-treated at 95 °C for a 10-min duration prior to electrophoresis. The expression of 2Gs/NV1 and 2Fs/NV2 was detected using anti-niv_G (AntibodySystem, Paris, France, #PVV07901) and anti-niv_F (AntibodySystem, Paris, France, #PVV08101) antibodies, respectively. Total protein lysates from untreated cells served as the mock control. To verify protein integrity, Beta-Actin was detected using an anti-Beta-Actin antibody (Proteintech, Wuhan, China, #HRP-60008) as an internal control.

### 2.7. Animal Immunization and Sample Collection

BALB/c mice and Syrian hamsters were immunized via intramuscular injection and divided into low-dose (2 µg) and high-dose (10 µg) groups, following a prime-boost regimen with a 14-day interval. Retroorbital sinus blood was collected using capillary tubes at 5 h, 24 h, 7 days, 14 days, 21 days, and 28 days post-initial immunization. Blood samples were collected in tubes without anticoagulant and centrifuged at 6000 rpm for 15 min at 4 °C. The separated serum was then stored at −80 °C for subsequent assays.

### 2.8. Antibody Endpoint Titer Measurement with ELISA

Recombinant F and G proteins (Antibodysystem, Paris, France, #EVV08101/#EVV07901) were immobilized onto 96-well plates (Thermo Fisher Scientific, Waltham, MA, USA, #442404) at a concentration of 1 µg/mL (16 h, 4 °C). Following thrice-repeated PBST (0.05% Tween-20) rinses, blocking was performed using 2% BSA in PBS (37 °C, 60 min). Serum harvested via orbital sinus venipuncture was serially diluted (1:100 initial dilution) in assay buffer and transferred to the microplates for primary antibody incubation (37 °C, 60 min). After removing unbound antibodies, secondary detection was carried out using HRP-labeled goat anti-mouse IgG (Invitrogen, Carlsbad, CA, USA, #A-10668, 1:30,000) or rabbit anti-hamster IgG (Cohesionbio, Suzhou, China, #CSA2182, 1:30,000) for 60 min at 37 °C. Washed wells were developed with TMB substrate (100 uL/well; Thermo Fisher Scientific, Waltham, MA, USA, #34028) for 15 min and quenched using SolarBio acidic stop reagent (SolarBio, Beijing, China, #C1058). Absorbance was recorded at 450 (primary) and 630 nm (reference).

Baseline negative controls comprised sera obtained from PBS-treated mice or Syrian hamsters, which were handled under identical conditions to the test samples. The positivity threshold was established at 2.1-fold the average optical density (OD) of the negative control wells. Antigen-specific IgG titers were quantified as the maximum dilution ratio that generated an absorbance reading surpassing this computed cutoff.

### 2.9. Pseudovirus Neutralization Assay

To eliminate complement activity, sera underwent thermal inactivation at 56 °C for 30 min. Assays were performed in 96-well plates containing cell controls (CCs) and virus controls (VCs). Following the addition of 100 µL DMEM to each experimental well, test sera (7.5 µL) were added to the first row (pre-filled with 42.5 µL DMEM) and subjected to a 3-fold serial dilution across six gradients. Pseudoviruses were thawed, adjusted to a predetermined concentration, and added (50 µL/well) to all experimental and VC wells. After a 1 h incubation at 37 °C, HEK 293T cells (100 µL at 5 × 10^5^ cells/mL) were seeded into each well. The plates were incubated at 37 °C for 48 h. Luminescence was quantified using the Bright-Glo Luciferase Assay System on a microplate reader. Neutralizing antibody titers were calculated and reported as the reciprocal of the serum dilution yielding a 50% inhibition.

### 2.10. Multiplex Cytokine Analysis in Serum

Whole-blood samples were harvested from the orbital vein of BALB/c mice at 5 h and 24 h post-initial immunization and subsequently centrifuged to isolate serum. Quantitative measurement of multiple cytokines was performed using the Bio-Plex Pro Mouse Cytokine 23-plex Assay kit (Bio–Rad, Shanghai, China, #64521678) on a Bio-Plex^®^ 200 System (Bio–Rad, Shanghai, China), strictly following the manufacturer’s instructions.

### 2.11. Enzyme-Linked Immunospot (ELISpot) Assay

ELISpot assays were executed following the standard instructions provided by the manufacturer (Mabtech, Nacka Strand, Sweden). Spleens were aseptically harvested from the mice or the Syrian hamsters and mechanically disaggregated through cell strainers to generate single-cell suspensions. Subsequently, mononuclear cells were isolated utilizing a lymphocyte isolation kit (Solarbio, Beijing, China, #P8860). To induce antigen-specific activation, cells were cultured with recombinant G, F proteins or G+F protein mixture (2 µg/50 µL per well). Unstimulated cells served as replicate-specific negative controls to measure baseline reactivity, whereas phytohemagglutinin (PHA)-stimulated cells acted as positive process controls.

Following plate equilibration at 37 °C under 5% CO_2_ and automated washing steps, cell suspensions were seeded into pre-coated 96-well ELISpot plates (Mabtech, Nacka Strand, Sweden; catalog numbers: mouse-IFNg, #3321-4APT–2; mouse–IL2, #3441–4APW–2; mouse–IL4, #3311–4APW–2; hamster–IFNg, #3102–4APW–2). High-resolution image capture and spot enumeration were performed utilizing the IRIS^TM^ Automated ELISpot Reader System (Mabtech, Nacka Strand, Sweden). Final results were normalized and reported as spot-forming units (SFU) per 10^6^ cells.

### 2.12. BCR/TCR Immune Repertoire

To yield DNA-free total RNA, whole-blood specimens were purified using the PAXgene Blood RNA System (Qiagen, Hilden, Germany, #762174) and treated with DNase I (Thermo Scientific, Waltham, MA, USA, #EN0521). RNA quality was verified via NanoDrop OneC, agarose gel electrophoresis, and Qubit 3.0 fluorometry. For library construction, 2 µg of total RNA was processed using the KC-Digital Stranded BCR/TCR-seq Library Prep Kit (Seqhealth Technology, Wuhan, China, #DT0815-02). Molecules were tagged with 8-nt unique molecular identifiers (UMIs) to mitigate PCR amplification biases. Following size selection (250–500 bp) and quantification, libraries were sequenced on an Illumina NovaSeq platform. Raw reads were filtered utilizing SOAPnuke (v1.6.0). For UMI-guided deduplication, reads with identical 8-nt UMIs and ≥95% sequence identity were clustered using custom scripts, generating a single error-corrected consensus read per cluster. Finally, V(D)J assignment and CDR3 identification were executed from the consensus reads using MiXCR (v3.0.3) mapped against the IMGT database.

### 2.13. Histopathological Evaluation

Tissue specimens were fixed in 4% paraformaldehyde (PFA) for 72 h prior to routine paraffin embedding. Serial sections (5 µm in thickness) were cut via a microtome and subjected to standard hematoxylin and eosin (H&E) staining. Whole-slide digital scanning was executed using a high-resolution 3DHISTECH platform (Pannoramic MIDI II, 3DHISTECH). Qualified veterinary pathologists evaluated the histopathology via computerized morphometric analysis within the CaseViewer 2.4.0 software (Budapest, Hungary), applying standardized semiquantitative scoring metrics to assess inflammatory cell infiltration and structural tissue modifications.

### 2.14. Statistical Analysis

All data are expressed as the mean ± SEM. Statistical evaluations were carried out utilizing GraphPad Prism 10.4.1 software (San Diego, CA, USA). For comparisons involving three or more cohorts, one-way ANOVA with Tukey’s post hoc test was applied for graphs. Statistical significance thresholds are designated as: * *p* < 0.05; ** *p* < 0.01; *** *p* < 0.001; **** *p* < 0.0001; ns, not significant.

## 3. Results

### 3.1. Design of the Head-to-Head Dimer-like Antigen and In Vitro Characterization of mRNA–LNPs

The G and F proteins of NiV are the primary targets for neutralizing antibodies and vaccine development. To optimize antigen design, we conducted a systematic analysis of B-cell and MHC epitopes for both proteins using the IEDB database (Figure S3). Our analysis revealed that the predominant immunogenic epitopes are highly concentrated within the globular head domains of both G and F proteins. This was further substantiated by antigen-antibody docking simulations with several previously characterized neutralizing antibodies, which confirmed that critical interaction sites are localized almost exclusively to the head domains (Figure S4).

**Figure 1:**
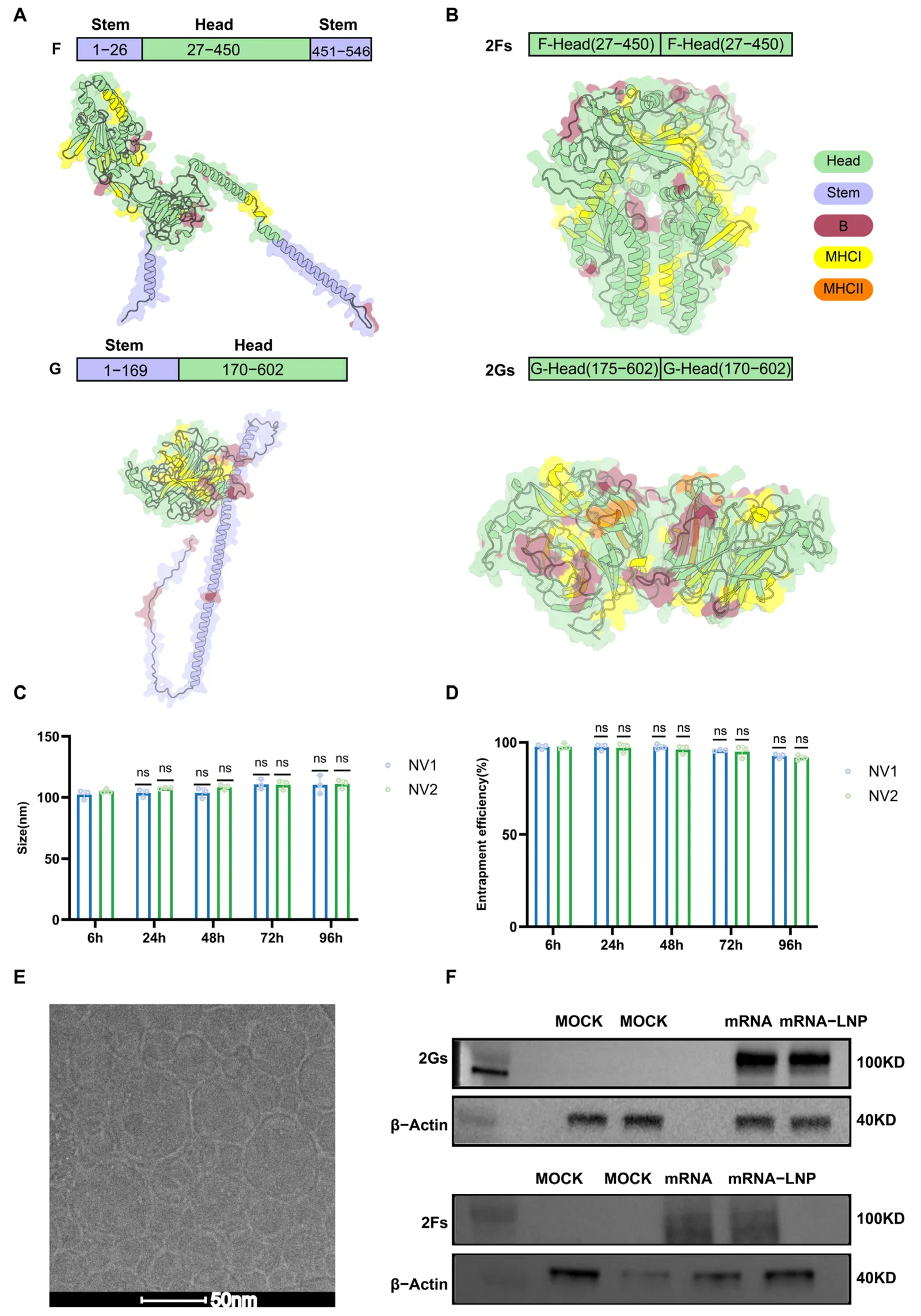
Design of the Head-to-Head Dimer–like Antigen and In Vitro Characterization of mRNA-LNPs. (**A**). Structural prediction and immunogenic epitope mapping of Antigen F/G. (**B**). Structural prediction and immunogenic epitope mapping of dimeric Antigen 2Fs/2Gs. (**C**). Particle size stability of mRNA-LNPs over 96 h. Significance was assessed by one-way ANOVA with Tukey’s post hoc test: ns, not significant (compared with 6 h). (**D**). Encapsulation efficiency stability of mRNA-LNPs over 96 h. (**E**). Cryo-EM images and morphological characterization of mRNA-LNPs. (**F**). Western blot indication of antigen expression in transfected HEK 293T cells. Significance was assessed by one-way ANOVA with Tukey’s post hoc test: ns, not significant (compared with 6 h).

To maximize epitope density and enhance immunogenicity, we engineered novel antigens, designated 2Gs and 2Fs, by excising the stalk domains and tandemly duplicating the epitope-rich head domains (Figure 1A,B). These constructs were then formulated into lipid nanoparticles (LNPs) to generate three mRNA vaccine candidates: NV1 (encoding 2Gs), NV2 (encoding 2Fs), and NV3 (a 1:1 mixture of NV1 vaccine and NV2 vaccine). Physicochemical characterization of the mRNA-LNPs demonstrated excellent stability over a 96 h period at 4 °C, with particle sizes maintained at approximately 100 nm and encapsulation efficiencies consistently exceeding 90%, and a near-neutral zeta potential. (Figure 1C,D, Figures S5 and S6). Cryo-electron microscopy (Cryo-EM) revealed that the mRNA-LNPs confirmed uniform spherical morphology with a characteristic solid core (Figure 1E). Finally, in vitro expression of the encoded antigens was confirmed via transfection into HEK 293T cells, followed by Western blotting (Figure 1F and Figure S7).

### 3.2. The mRNA Vaccines NV1 and NV3, Both Containing Antigen 2Gs, Were Observed to Elicit More Robust Humoral Immune Responses

**Figure 2:**
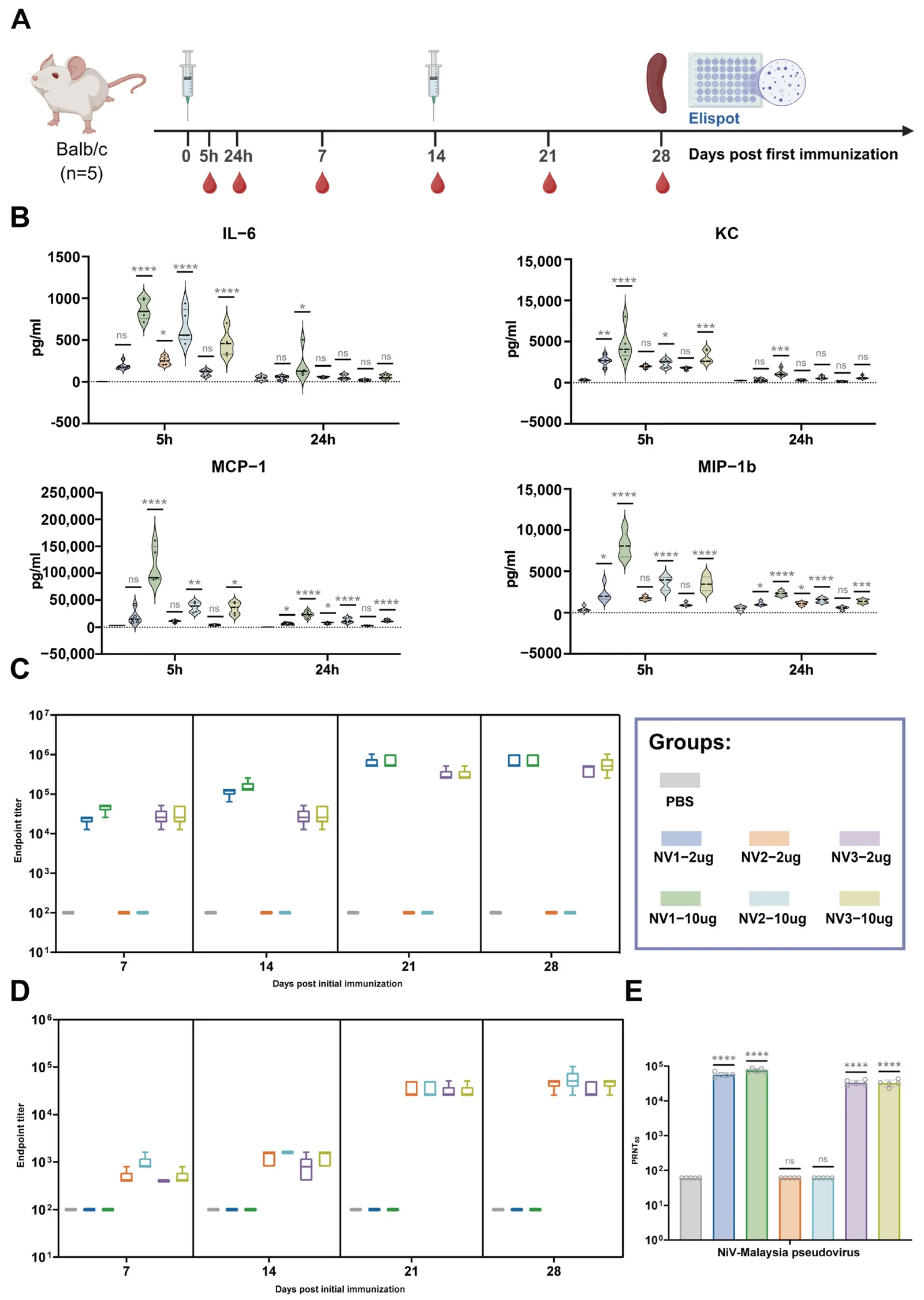
The mRNA vaccines NV1 and NV3, both containing antigen 2Gs, were observed to elicit more robust humoral immune responses. (**A**). Schematic of the immunization schedule and experimental design in BALB/c mice. (**B**). Quantitative analysis of the secretion dynamics of immunomodulatory cytokines IL 6/KC/MCP-1/MIP-1b in serum at 5 h and 24 h post-initial immunization. Bars indicate mean ± SEM; significance was assessed by one-way ANOVA with Tukey’s post hoc test: ns, not significant, *p*  >  0.05; * *p*  <  0.05; ** *p*  <  0.01; *** *p*  <  0.001; and **** *p*  <  0.0001 (compared with PBS group). (**C**). G-specific binding antibody titers in mouse sera at various time points post-initial immunization. Bars indicate mean ± SEM. (**D**). F–specific binding antibody titers in mouse sera at various time points post-initial immunization. Bars indicate mean ± SEM. (**E**). Neutralizing antibody titers against NiV Malaysia pseudovirus in mouse sera at 28 days post-initial immunization. Bars indicate mean ± SEM; significance was assessed by one-way ANOVA with Tukey’s post hoc test: ns, not significant, *p*  >  0.05 and **** *p*  <  0.0001 (compared with PBS group).

To evaluate the immunogenicity of the engineered antigens, we established three vaccine groups: NV1 (encoding 2Gs), NV2 (encoding 2Fs), and NV3 (a 1:1 mixture of NV1 vaccine and NV2 vaccine). BALB/c mice were immunized intramuscularly with either low (2 µg) or high (10 µg) doses following a prime-boost regimen with a 14 day interval (Figure 2A).

To assess the early innate immune activation, serum was collected at 5 h and 24 h post-initial immunization for multiplex cytokine analysis. The results showed that several pro-inflammatory and chemotactic cytokines were significantly upregulated at 5 h compared to the PBS control group, reflecting the rapid immune mobilization characteristic of mRNA-LNP platforms (Figure 2B) [31,32]. Specifically, IL-6 levels were elevated, potentially promoting B-cell differentiation into plasma cells. Circulating levels of the chemokines KC (CXCL1) and MCP-1 (CCL2) were markedly increased following intramuscular administration, typically reflecting a transient systemic mobilization profile associated with neutrophils and monocytes/antigen-presenting cells (APCs), respectively, which are key drivers for initiating adaptive immunity. Additionally, elevated serum MIP-1b (CCL4) levels indicated broad systemic innate immune activation, typically creating a cytokine environment supportive of natural killer (NK) cell and pro-inflammatory Th1 responses. Crucially, cytokine levels at 24 h were markedly lower than those at 5 h, indicating a transient and controlled innate response, which supports a favorable safety profile (Figure 2B and Figure S8) [33]. Furthermore, detailed pairwise statistical comparisons between different vaccinated groups are provided in Figure S9.

Binding antibody kinetics were monitored throughout the study. All three vaccine candidates elicited detectable binding antibodies as early as 7 days post-prime, with titers significantly amplified following the booster dose, demonstrating effective booster-enhanced antibody responses (Figure 2C,D). Notably, NV2 (encoding 2Fs) generated lower binding antibody levels compared to NV1 (encoding 2Gs) and NV3 (a 1:1 mixture of NV1 vaccine and NV2 vaccine). A similar trend was observed in pseudovirus neutralization assays: both NV1 and NV3 induced potent neutralizing antibody (nAb) responses. In contrast, NV2 failed to elicit detectable neutralizing activity, despite achieving binding antibody titers as high as 10^5^. This discrepancy likely stems from the 2Fs antigen’s inability to maintain the metastable pre-fusion conformation required for the exposure of critical neutralizing epitopes (Figure 2E).

### 3.3. The mRNA Vaccines NV2 and NV3, Both Containing Antigen 2Fs, Were Observed to Elicit More Robust Cellular Immune Responses

**Figure 3:**
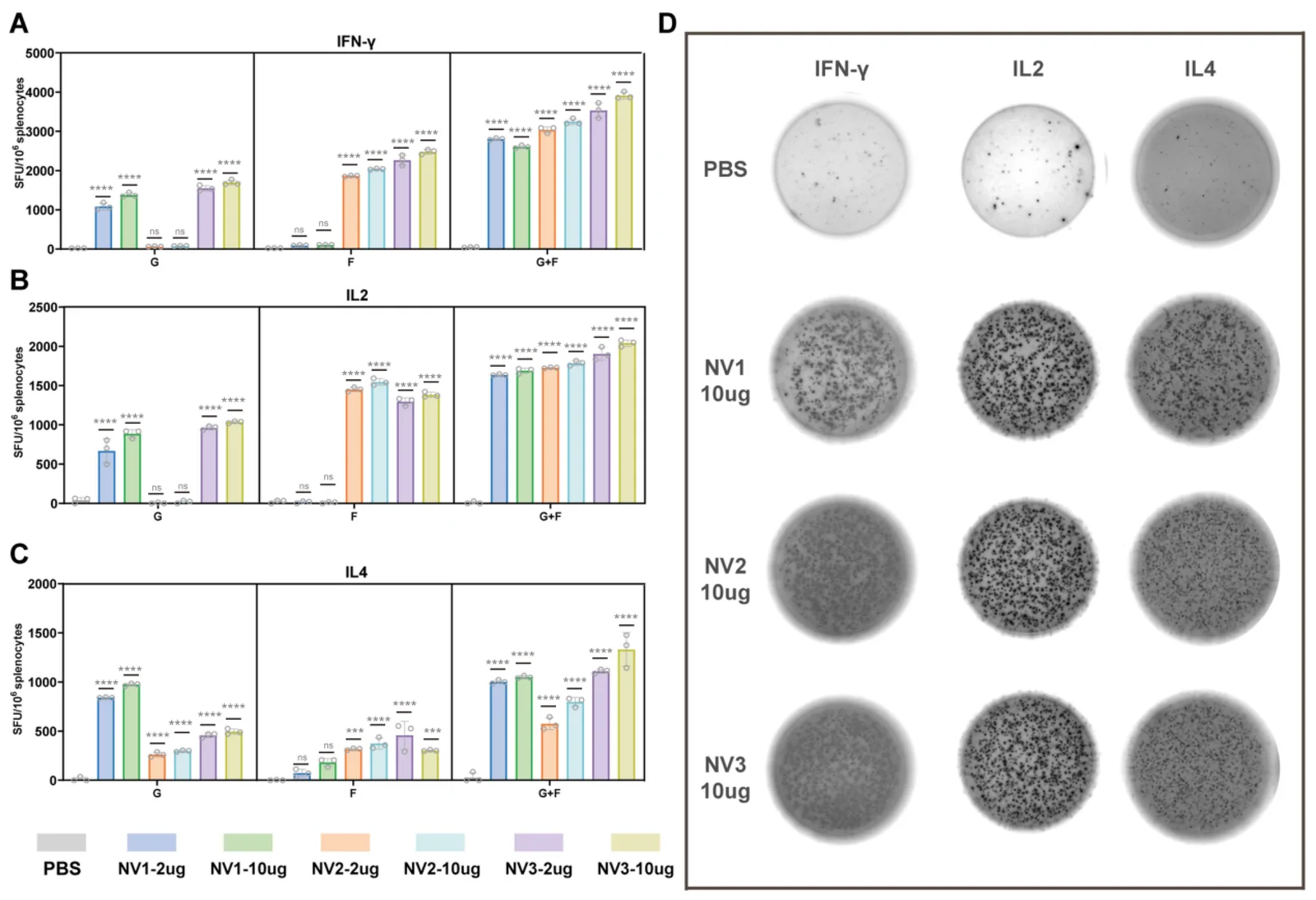
The mRNA vaccines NV2 and NV3, both containing antigen 2Fs, were observed to elicit more robust cellular immune responses. (**A**). IFN-γ secretion levels in response to G protein, F protein and G+F protein at 28 days post-initial immunization. Bars indicate mean ± SEM; significance was assessed by one-way ANOVA with Tukey’s post hoc test: ns, not significant, *p*  >  0.05 and **** *p*  <  0.0001 (compared with PBS group). (**B**). IL-2 secretion levels in response to G protein, F protein and G+F protein at 28 days post-initial immunization. Bars indicate mean ± SEM; significance was assessed by one-way ANOVA with Tukey’s post hoc test: ns, not significant, *p*  >  0.05 and **** *p*  <  0.0001 (compared with PBS group). (**C**). IL-4 secretion levels in response to G protein, F protein and G+F protein at 28 days post-initial immunization. Bars indicate mean ± SEM; significance was assessed by one-way ANOVA with Tukey’s post hoc test: ns, not significant, *p*  >  0.05; *** *p*  <  0.001 and **** *p*  <  0.0001 (compared with PBS group). (**D**). Representative ELISpot images showing cytokine-producing cell spots.

To systematically evaluate whether the vaccine antigens could activate cell-mediated immunity, we performed ELISpot assays on splenocytes isolated from vaccinated BALB/c mice. To adequately simulate recall immune responses, splenocytes were stimulated ex vivo with recombinant NiV F protein, G protein, or an F+G protein mixture to quantify the secretion of IFN-γ (Figure 3A and Figure S10), IL-2 (Figure 3B and Figure S11), and IL-4 (Figure 3C,D, Figures S12 and S13). Statistical significance indicated in Figure 3 corresponds to comparisons between each vaccinated group and the PBS control group. To maintain clarity while providing full statistical evaluations, complete pairwise comparisons across all experimental groups are presented in Figure S14.

The results demonstrated that all three vaccine candidates successfully elicited detectable cytokine secretion. Specifically, NV2 (encoding 2Fs) and the bivalent NV3 (a 1:1 mixture of NV1 vaccine and NV2 vaccine) induced substantially higher levels of IFN-γ and IL-2 compared to NV1 (encoding 2Gs). In contrast, while NV1 elicited higher levels of IL-4 secretion, these responses remained relatively low in magnitude when compared to the production of IFN-γ and IL-2. Collectively, the cytokine secretion profiles of all three vaccines exhibited a predominant Th1-biased response. This immunological polarization may be attributed to the endogenous antigen presentation pathway facilitated by the mRNA platform, as well as the intrinsic adjuvant properties of the lipid nanoparticle (LNP) delivery system.

### 3.4. The mRNA Vaccines Exhibit Distinct BCR/TCR Immunological Characteristics

Based on the systematic immunogenicity evaluation described above, we observed that vaccines containing the Gs antigen elicited superior humoral immune responses, characterized by high titers of binding antibodies and pseudovirus-neutralizing antibodies. Conversely, vaccines containing the Fs antigen demonstrated enhanced cellular immune responses, specifically manifesting as a distinct Th1-biased cellular immunity.

**Figure 4:**
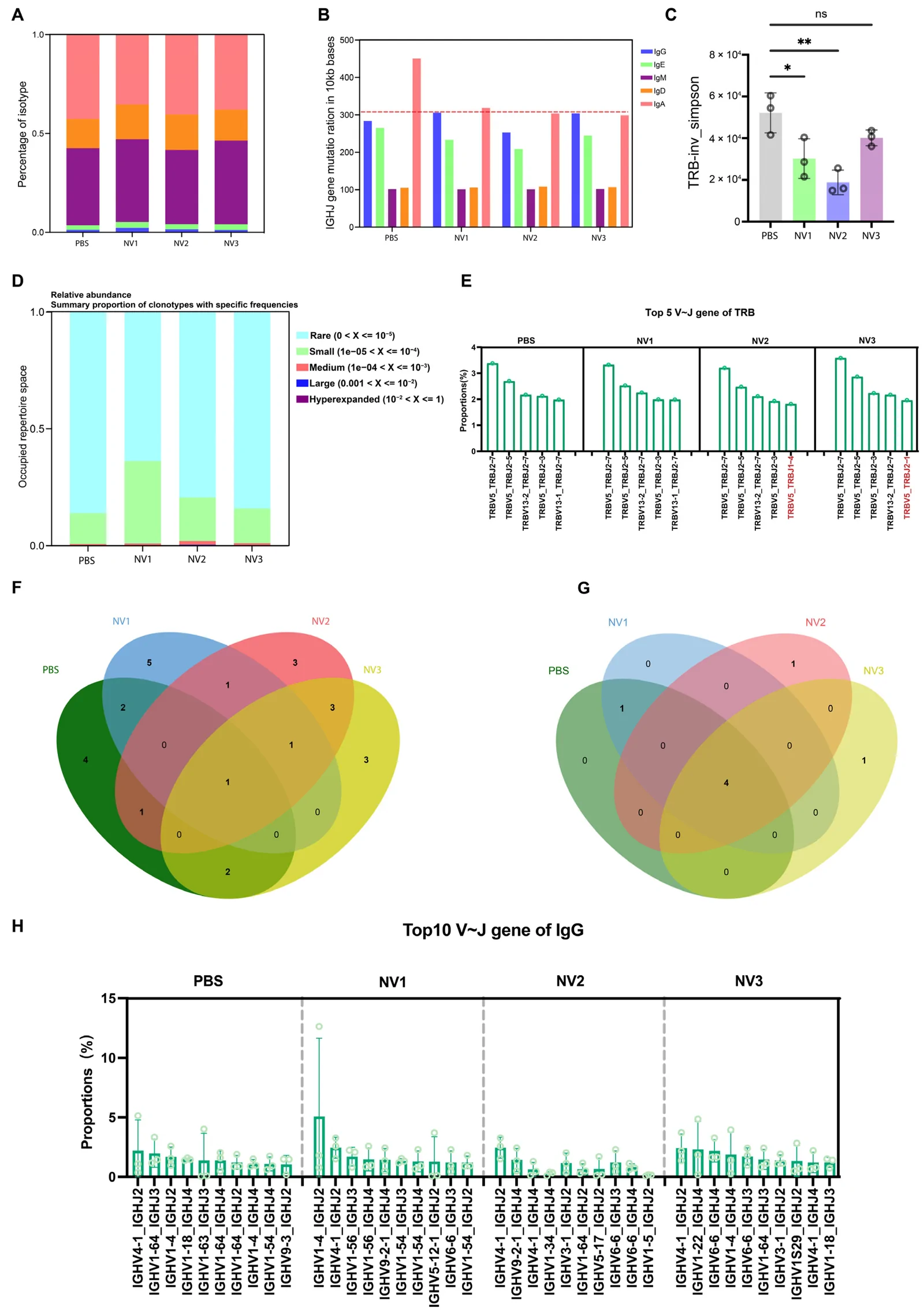
The mRNA vaccines exhibit distinct BCR/TCR immunological characteristics. (**A**). Repertoire composition of IgH across different groups. (**B**). Somatic hypermutation (SHM) patterns of IgH J genes across different groups. (**C**). Inverse Simpson index of TRB repertoires across different groups. Bars indicate mean ± SEM; significance was assessed by one-way ANOVA with Tukey’s post hoc test: ns, not significant, *p*  >  0.05; * *p*  <  0.05 and ** *p*  <  0.01. (**D**). Clonotype distribution of TRB across different groups. (**E**). Top 5 V-J gene pairings in TRB repertoires. (**F**). Similarity analysis of the top 10 V-J gene pairings in IgG across groups. (**G**). Similarity analysis of the top 5 V-J gene pairings in TRB across groups. (**H**). Top 10 V-J gene pairings in IgG repertoires. Bars indicate mean ± SEM.

To investigate the immune mechanisms underlying these divergent immunogenicity profiles, we performed BCR and TCR sequencing on the vaccinated mice. The sequencing results revealed a similar distribution of immunoglobulin heavy-chain (IGH) isotypes across all groups, predominantly consisting of IgA and IgM. However, the proportion of IgG in the Gs vaccine group was higher compared to the other three groups (Figure 4A). Consistent with this finding, we observed a similar trend in the analysis of IGHV/IGHJ gene mutation frequencies, where the proportion of IgG was elevated in the Gs and GsFs groups (Figure 4B). Furthermore, the proportion of IgE-expressing B cells was significantly higher in NV1- and NV3-immunized mice than in PBS controls. These data suggest that the humoral immune responses elicited by these two vaccines may be biased toward Th2/IgE class switching (Figure S15).

Furthermore, we analyzed the usage frequency of the top 10 V-J gene pairings for both IgG and IgM. The analysis revealed a distinct pattern: while the other three groups predominantly utilized IGHV4-1/IGHJ2, the Gs group exhibited the highest usage frequency for the IGHV1-4/IGHJ2 pairing (Figure 4H). These marked differences in the top V-J gene repertoire signatures indicate that the three vaccine candidates activate distinct BCR immune features, which appears to be a critical factor underlying the observed disparities in humoral immunity.

Regarding the T-cell receptor (TCR) repertoire, we found that the inverse Simpson index in all vaccine groups was lower than that of the control group, signifying the induction of antigen-driven oligoclonal expansion. Compared to the other groups, the Fs group exhibited the lowest inverse Simpson index, suggesting that it triggered a more extensive T-cell clonal proliferation (Figure 4C). Additionally, the clonotype distribution in the Fs group differed from the other three groups, characterized by an increased proportion of Small, Medium, and Large clones, further corroborating the formation of significant oligoclonality (Figure 4D). We also examined the top 5 TRB V-J gene usage frequencies (Figure 4E). While there were no major global differences between the vaccine and control groups, it is noteworthy that the Fs and GsFs groups displayed a specific V-J gene pairing (TRBV5/TRBJ1-4 or TRBV5/TRBJ2-1) that was absent from the top 5 pairings of the other groups (Figure 4G).

In summary, our analysis of the BCR and TCR repertoires identified distinct immune signatures associated with each candidate. These differential adaptive immune profiles offer a compelling mechanistic explanation for the varying immunogenic characteristics observed across the different vaccine groups (Figures S16 and S17).

### 3.5. The mRNA Vaccines Demonstrated an Acceptable Safety Profile, with No Significant Abnormalities in Indicators or Organ Damage Observed

Although mRNA vaccines are recognized for their robust efficacy and rapid mobilization of immune responses, safety remains a paramount public concern. To characterize the safety profile of our candidates, we conducted a comprehensive evaluation of NV1 (encoding 2Gs), NV2 (encoding 2Fs), and NV3 (a 1:1 mixture of NV1 vaccine and NV2 vaccine). We monitored body temperature and body weight in BALB/c mice for 5 days post-intial immunization. On day 5, serum and vital organs were harvested for biochemical analysis and histopathological evaluation.

**Figure 5:**
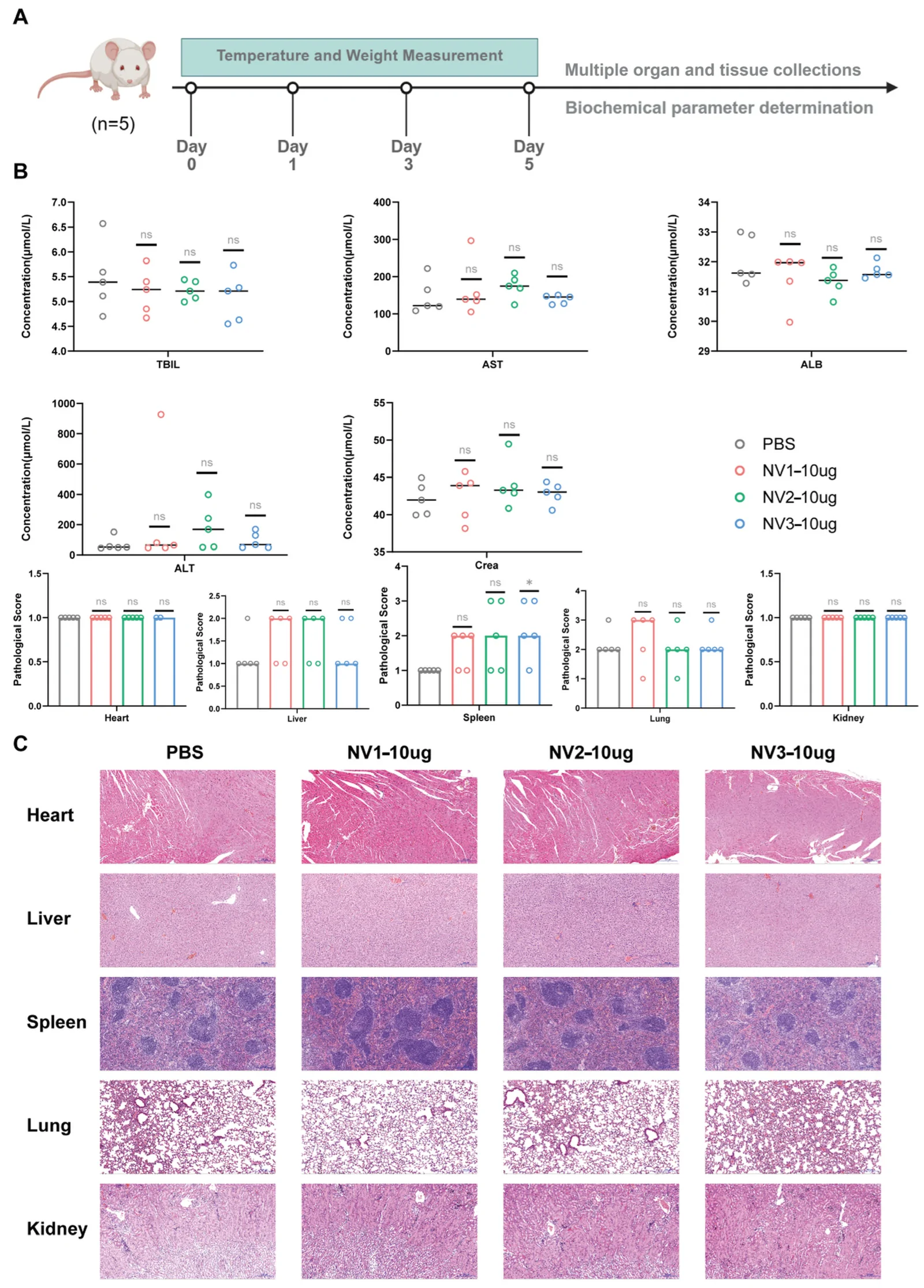
The mRNA vaccines demonstrated an acceptable safety profile, with no significant abnormalities in indicators or organ damage observed. (**A**). Serum biochemical parameters, including TBIL, AST, ALT, CREA, and ALB. (**B**). Histopathological scoring of major organs across different groups. Bars indicate mean ± SEM; significance was assessed by one–way ANOVA with Tukey’s post hoc test: ns, not significant, *p*  >  0.05 and * *p*  <  0.05. (**C**). Representative H&E-stained sections of major organs (Scale bar: 200 µm).

The results demonstrated that vaccination had no adverse impact on body temperature or weight, with growth trends consistent with those of the PBS control group (Figures S18 and S19). Functional biomarkers, including ALT and AST, showed no significant elevation, indicating preserved hepatic function (Figure 5A). Furthermore, histopathological analysis of the heart, liver, lungs, and kidneys revealed no evidence of organ damage or inflammatory injury compared to the control group (Figure 5B,C).

Notably, we observed that pathological scores in the spleen of vaccinated mice were moderately higher than those in the control group. This was primarily characterized by the formation of active germinal centers and localized inflammatory cell infiltration. Rather than indicating toxicity, these splenetic changes are consistent with potent vaccine-induced immune activation and lymphocyte proliferation. Collectively, these preliminary safety findings suggest that all three mRNA vaccine candidates possess a favorable safety profile for further development.

### 3.6. The Vaccines Elicit a Measurable Immune Response in Syrian Hamsters

**Figure 6:**
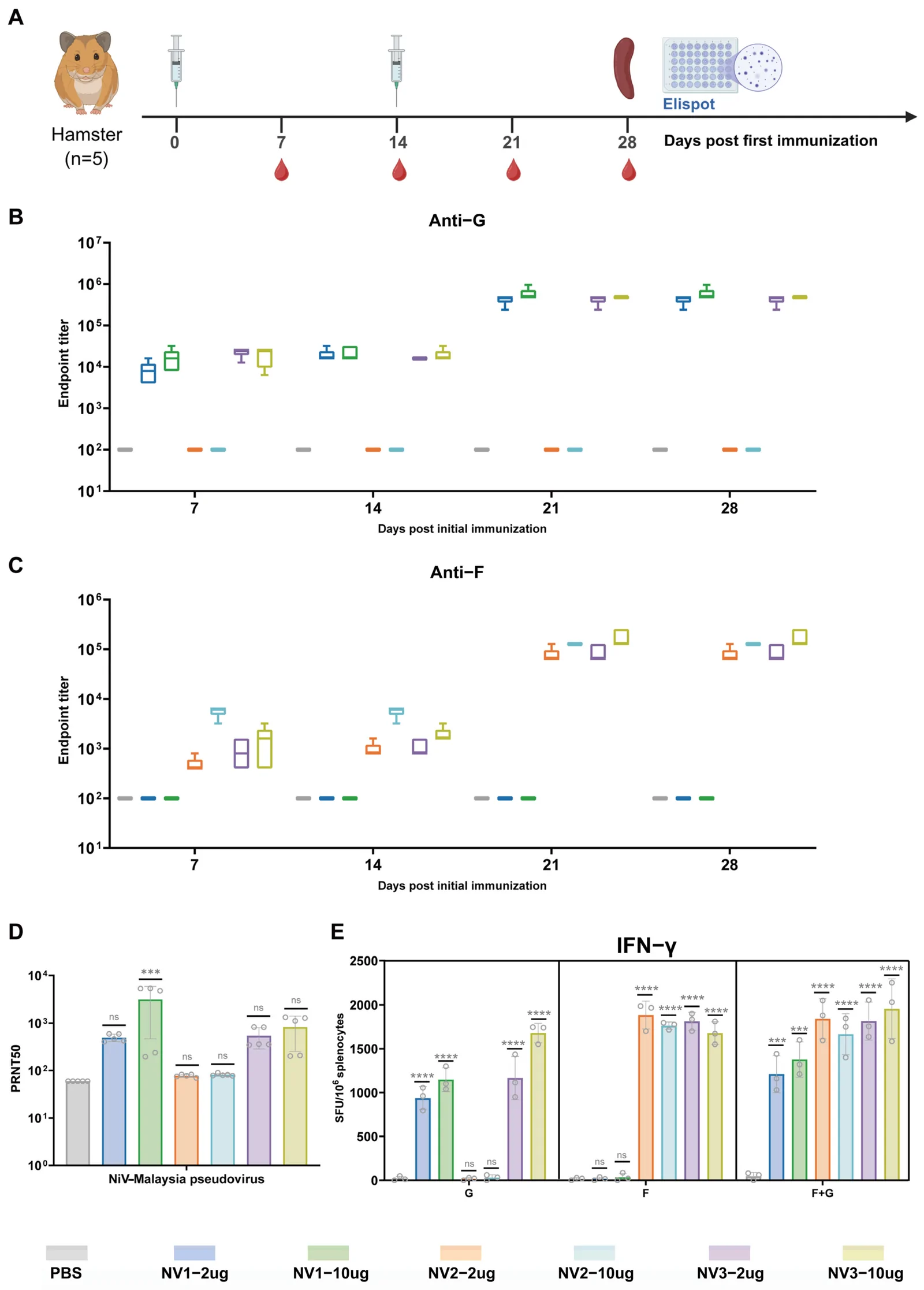
The vaccines elicits a measurable immune response in Syrian hamsters. (**A**). Schematic of the experimental design and immunization schedule in Syrian hamsters. (**B**). G-specific binding antibody titers in hamster sera at indicated time points post-initial immunization. Bars indicate mean ± SEM. (**C**). F-specific binding antibody titers in hamster sera at indicated time points post-initial immunization. Bars indicate mean ± SEM. (**D**). Neutralizing antibody titers against NiV pseudovirus at 28 days post-initial immunization. Bars indicate mean ± SEM; significance was assessed by one-way ANOVA with Tukey’s post hoc test: ns, not significant, *p*  >  0.05 and *** *p*  <  0.001 (compared with PBS group). (**E**). IFN-γ secretion levels in response to different stimulants at 28 days post-initial immunization. Bars indicate mean ± SEM; significance was assessed by one–way ANOVA with Tukey’s post hoc test: ns, not significant, *p*  >  0.05; *** *p*  <  0.001 and **** *p*  <  0.0001 (compared with PBS group).

Syrian hamsters are susceptible to infections by various viruses, including Nipah virus, and are therefore widely used to evaluate the challenge protection efficacy of vaccines or antiviral agents. With the aim of further exploring protective efficacy, the immunogenicity profiles of the three mRNA constructs were evaluated in hamsters utilizing identical dosing regimens and administration pathways (Figure 6A). The results demonstrated that all three vaccines induced detectable immune responses. Specifically, each vaccine elicited detectable levels of binding antibodies (Figure 6B,C). Furthermore, all three candidates elicited IFN-γ secretion to varying extents, indicating the induction of cellular immune responses (Figure 6E, Figures S13 and S20). In pseudovirus neutralization assays, only the 10 µg dose of NV1 elicited a statistically significant neutralizing antibody response (Figure 6D), whereas NV2 and NV3 showed minimal or no significant neutralizing activity, which was consistent with the observations in BALB/c mice. In summary, all three vaccine candidates induced measurable immune responses with distinct characteristics in Syrian hamsters, offering valuable insights into Nipah virus antigen design that warrant further investigation.

## 4. Discussion

Outbreaks of Nipah virus (NiV) infection have occurred in multiple regions worldwide. Given its high fatality rate and pathogenicity, NiV poses a serious threat to global public health security. Although several candidate vaccines have entered the clinical trial phase, no clinically approved prophylactic vaccine is currently available [34,35,36,37].

The NiV G and F glycoproteins are essential for viral entry and represent the primary targets for vaccine development. Numerous studies have demonstrated that the G glycoprotein can elicit neutralizing antibodies [38,39] and the immunogenicity of the F glycoprotein is highly dependent on its prefusion conformation (pre-F) [40,41]. However, the full immunogenic potential, specific characteristics and dominant antigenic epitopes of both glycoproteins require further investigation.

In contrast to conventional vaccine candidates that employ full-length G and F glycoproteins, our antigen design adopted an “immunofocusing” strategy [42,43]. By removing the immunologically subdominant stalk domain, we redirected the humoral immune response toward the head domain, which harbors a greater number of neutralizing epitopes. This approach increased the proportion of antibodies targeting potentially neutralizing sites and enhanced the overall precision of the antibody response. In addition, tandem repeats of the head domain were introduced to exploit multivalent display effects, thereby promoting B-cell receptor (BCR) crosslinking and aggregation and strengthening B-cell activation signals. These findings are consistent with the high titers of pseudovirus-neutralizing antibodies observed with NV1 (encoding 2Gs).

Previous studies have developed polymeric recombinant protein vaccines based on monomers and ferritin nanoparticles. These vaccines utilize the G-head domain and significantly enhance immunogenicity through the multivalent presentation provided by ferritin nanoparticles [44]. Compared with previously reported ferritin nanoparticle-based G-head vaccines, our G-head domain pseudo-dimer antigen elicited higher G–specific antibody titers in a two-dose prime-boost regimen at equivalent dosing. These results further support the applicability and advantage of combining epitope-focused targeting with pseudo-dimer multivalency in vaccine antigen design.

In addition to these approaches, the Nipah virus vaccine field has yielded several other advanced immunogen strategies, including highly engineered constructs such as stabilized pre-F/G chimeric trimers [17] and self-assembling mRNA-encoded nanoparticles displaying 60 G–head domains (mRNA NiV G-NP) [45]. In contrast, our tandem head-to-head dimer antigens represent a more streamlined design. This strategy avoids the potential assembly bottlenecks and complex manufacturing demands associated with macromolecular constructs, while also eliminating the need for nanoparticle-mediated multivalent display and the reliance on intricate intracellular self-assembly and post-translational folding dynamics.

Notably, although NV2 (encoding 2Fs) failed to induce detectable pseudovirus-neutralizing antibodies, it elicited a strong Th1-biased cellular immune response, which plays a critical role in viral clearance. The absence of neutralizing antibodies against the F glycoprotein may be attributable to the inability of the NV2 antigen (2Fs) to maintain the prefusion conformation of F, resulting in the loss of conformation-dependent neutralizing epitopes. The F glycoprotein is a class I transmembrane protein with highly unstable conformation; thus, the truncated 2Fs antigen, lacking support from the stalk and transmembrane domains, struggles to form a stable prefusion state [40,41]. Future efforts could involve designing trimerization or introducing mutations to stabilize the prefusion conformation, thereby better exploiting the immunogenic potential of the F glycoprotein. Studies have shown that the prefusion conformation of F induces higher neutralizing antibody titers compared to the postfusion form. For instance, immunization of mice with sFGCNt (prefusion-stabilized) elicited stronger neutralizing antibody responses than non-GCNt-appended sF [46], further confirming the critical importance of the F prefusion conformation in inducing neutralizing antibodies. The bivalent vaccine NV3, obtained by mixing NV1 vaccine and NV2 vaccine at a 1:1 ratio, successfully induced robust humoral and cellular immune responses. This dual-antigen strategy compensates for the immunogenic deficiencies of individual antigens, achieving more comprehensive immune activation and suggesting that bivalent vaccines may represent a superior approach for NiV vaccine design, providing new insights for subsequent development.

Regarding systemic safety, we evaluated the safety and immunogenicity of the candidate vaccines in BALB/c mice. The results showed no significant elevations in major biochemical parameters and no adverse pathological changes in vital organs (heart, liver, lungs, and kidneys), confirming the low toxicity profile of our vaccines. Notably, splenic pathological scores in vaccinated mice were slightly higher than those in controls. This observation is interpreted as a histological hallmark of potent immune activation. The formation of active germinal centers and local inflammatory cell infiltration indicate vigorous lymphocyte proliferation and antigen-driven immune activation in secondary lymphoid organs. These morphological changes align closely with our B-cell receptor (BCR) and T-cell receptor (TCR) sequencing data. Collectively, these findings demonstrate that the candidate vaccines effectively mobilize the immune system while coordinating efficient immune responses under conditions of systemic safety.

In addition, the immunogenicity profiles observed in the Syrian hamster model were highly consistent with trends in the BALB/c mouse model, demonstrating remarkable cross-species concordance. These results underscore the potential of these vaccines to confer protection in highly susceptible models, laying a solid foundation for subsequent efficacy studies and clinical translation.

Despite these encouraging results, several limitations must be acknowledged. Although the candidate vaccines exhibited strong immunogenicity, the strict biosafety level 4 (BSL-4) requirements for live NiV severely restricted our ability to perform live-virus neutralization assays and challenge studies at this stage. Additionally, an apparent discrepancy was observed between our immune repertoire sequencing and serological data. While BCR sequencing revealed a higher proportion of IgG transcripts in the NV1 group compared to the NV3 group, both groups elicited similar binding and neutralizing antibody titers. We hypothesize that other antibody isotypes may contribute significantly to the robust humoral response observed with the NV3 bivalent vaccine; however, the lack of comprehensive antibody isotype profiling in the present study limits our ability to fully verify this underlying mechanism. Moreover, current in vitro characterization via denaturing Western blot is limited to primary structure, leaving the native conformation, glycosylation, and lipid association of the expressed antigens to be fully elucidated. Furthermore, although our epitope–guided and structure-guided designs were indicated through high-precision computational simulations, the precise conformational arrangements of the 2G and 2F dimers have not yet been empirically confirmed using high-resolution techniques such as cryo-electron microscopy (cryo-EM). Addressing these technical gaps will be a priority in future research to further enhance the translational potential of these candidate vaccines.

## 5. Conclusions

This study illustrates the application of an epitope- and structure-guided “immune-focusing” strategy for developing novel Nipah virus mRNA–LNP vaccine candidates. By engineering head–to–head dimeric antigens (2Gs and 2Fs), we demonstrated that distinct antigen designs can effectively shape specific adaptive immune trajectories. Specifically, NV1 drove robust humoral immunity with potent neutralizing activity, whereas NV2 stimulated a distinct Th1–biased cellular response. NV3 (1:1 mixed formulation) successfully integrated these features to elicit a balanced humoral and cellular immune response. Supported by deep immune repertoire sequencing and favorable safety profiles in both murine and Syrian hamster models, these candidates provide valuable immunological insights. Although further protective efficacy studies involving live-virus challenges are warranted to determine the optimal prophylactic strategy, this rational antigen design framework contributes to the development of a versatile platform for NiV and other highly pathogenic paramyxoviruses.

## Data Availability

The data generated or analyzed during the current study are available from the corresponding authors upon reasonable request.

## Supplementary Materials

The following supporting information can be downloaded at https://www.mdpi.com/article/10.3390/vaccines14080718/s1, Figure S1: The mRNA sequence of NV1-encoding 2Gs as the antigen; Figure S2: The mRNA sequence of NV2-encoding 2Fs as the antigen; Figure S3: B-cell Linear Epitopes and MHC Epitopes of NiV G/F Analyzed Using the IEDB Database; Figure S4: Docking prediction of the NiV G/F protein in complex with the corresponding antibody, performed using AlphaFold3; Figure S5: Characterization of NV1 mRNA-LNPs; Figure S6: Characterization of NV2 mRNA-LNPs; Figure S7: Full-length Western blot membranes showing the demonstration of protein expression in vitro; Figure S8: Heatmap showing the dynamic changes in serum cytokine levels at 5 h and 24 h post-initial immunization (*n* = 5). Data are log2-transformed; log2FC (fold change) represents the ratio of cytokine levels at 5 h relative to 24 h post-initial immunization; Figure S9: Pairwise statistical comparisons of serum cytokine levels across experimental groups at 5 h and 24 h post-initial immunization; Figure S10: Representative IFN-γ ELISpot images from the cellular immunogenicity assessment; Figure S11: Representative IL-2 ELISpot images from the cellular immunogenicity assessment; Figure S12: Representative IL-4 ELISpot images from the cellular immunogenicity assessment; Figure S13: Representative ELISpot images and spot counts using PHA stimulation as a positive control; Figure S14: Statistical significance analysis of ELISpot assays using different stimuli in immunized BALB/c mice; Figure S15: Statistical analysis of antibody isotype (IgG/IgM/IgA) proportions within IGHV gene families in BCR-seq data. Bars indicate mean ± SEM; significance was assessed by one-way ANOVA with Tukey’s post hoc test; Figure S16: CDR3 length distribution and conserved CDR3 sequences across different immunization groups; Figure S17: Top 10 V-J gene pairing frequencies in IgM and IgD repertoires across different groups; Figure S18: Body temperature changes of BALB/c mice on days 1–5 post initial immunization; Figure S19: Body weight changes of BALB/c mice on days 1–5 post initial immunization; Figure S20: Statistical significance analysis among groups in the hamster ELISpot assay.

## Abbreviations

The following abbreviations are used in this manuscript:

WHO	World Health Organization
NiV	Nipah virus
G	Glycoprotein
F	Fusion
TCR	T-cell receptor
BCR	B-cell receptor
IACUC	Institutional Animal Care and Use Committee
DMEM	Dulbecco’s modified Eagle’s medium
FBS	Fetal bovine serum
SPF	Specific pathogen-free
PFA	Paraformaldehyde
cryo-EM	Cryo-electron microscopy
ELISA	Enzyme-linked immunosorbent assay
ELISpot	Enzyme-linked immunospot assay
LNP	Lipid nanoparticle
mRNA	Messenger RNA
TCID_50_	Median tissue culture infectious dose
UTR	Untranslated region
SD	Standard Deviation
CFR	Case fatality rate
ARDS	Acute respiratory distress syndrome
nAb	Neutralizing antibody
LNP/LNPs	Lipid nanoparticle/nanoparticles
MHC	Major histocompatibility complex
IEDB	Immune Epitope Database
PDB	Protein Data Bank
EE	Encapsulation efficiency
TEM	Transmission electron microscopy
TMB	3,3′,5,5′-tetramethylbenzidine
UMI/UMIs	Unique molecular identifier/identifiers
APCs	Antigen-presenting cell/cells
NK	Natural killer
Th1	T helper 1
IFN-γ	Interferon-gamma
IGH	Immunoglobulin heavy chain
TRB	T-cell receptor beta chain
ALT & AST	Alanine aminotransferase & aspartate aminotransferase
pTM	Predicted Template Modeling score
