# Supplementary material for: Epitope-Based Nipah Virus G and F Head-to-Head Dimer mRNA Vaccines Exhibit Distinct Immunogenicity and Immune Profiles
Source: Vaccines (Basel). 2026 Aug 20;14(8):718. doi: 10.3390/vaccines14080718 (PMC13517585; doi:10.3390/vaccines14080718)
Supplement: Supplementary file 1 [file vaccines-14-00718-s001.zip › vaccines-4458997-supplementary.pdf]

Figure S1 The mRNA sequence of NV1 encoding 2Gs as the antigen.

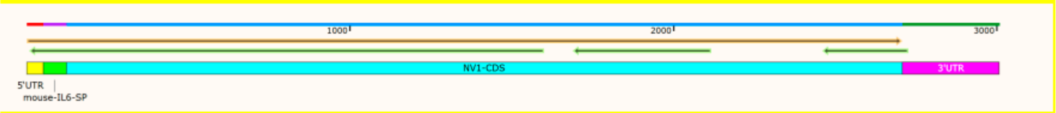

GAAUAAACUAGUAUUCUUCUGGUCCCCACAGACUCAGAGAGAACCCGCCACCAUGAAAUUUCUGAGCGCUAGGGACUU
UCACCCAGUGGCUUUCCUGGGCCUGAUGCUGGUGACCACCACCGCCACAGAGGGCGUGAGCAACCUGGUGGCCUGCC
CAAUAAUAUCUGCCUGCAGAAAACAAGCAAUCAGAUCCUGAAGCCAAAGCUGAUCUCUUAUACCCUGCCCGUGGUGGG
CCAGAGCGGCACCUGUAUCACUGAUCCCCUGCUGGCCAUGGAUGAGGGCUACUUUGCCUACAGCCACCUGGAGAGGAU
CGGCAGCUGCAGCAGAGGCGUGAGCAAGCAGAGGAUCAUCGGAGUGGGGAAGUGCUGGAUAGAGGAGAUGAGGUG
CCUAGUCUGUUUAUGACAAACGUGUGGACACCUCCAAAUCCUAACACCGUGUACCACUGUAGCGCAGUGUAUAAUAAU
GAGUUUUACUACGUGCUGUGCGCUGUGAGCACUGUGGGGGACCCUAUUCUGAAUAGUACCUACUGGAGCGGCAGCCU
GAUGAUGACCAGACUGGCCGUGAAGCCUAAGUCUAAUGGCGGAGGCUACAACCAGCAUCAGCUGGCUCUGCGGAGCAU
UGAGAAGGGAAGAUACGAUAAAGUGAUGCCUUACGGCCCAAGCGGGAUCAAACAGGGCGAUACCCUCUACUUCCCAGC
UGUGGGCUUUCUGGUGAGAACAGAGUUUAAGUAUAAUGACUCUAAUUGCCCUAUCACAAAAUGCCAGUACUCUAAAC
CCGAAAACUGUAGACUGAGCAUGGGCAUCAGACCUAAUUCCCAUUACAUCCUGAGAUCUGGCCUGCUGAAGUACAAUC
UGUCCGACGGGGAAAAUCCCAAGGUUGUUUCAUUGAGAUUAGCGACCAGAGACUGUCCAUCGGCUCCCCUUCUAAG
AUCUAUGACAGCCUGGGCCAGCCCGUGUUCUACCAGGCCAGCUUUAGUUGGGAUACAAUGAUUAAGUUGGCGAUGU
GCUGACCGUGAAUCCACUCGUGGUGAACUGGCGGAAUAAUACCGUGAUCUCUAGACCUGGCCAGAGCCAGUGCCCAAG
GUUCAACACCUGUCCUGAGAUCUGCUGGGAGGGCGUGUAUAACGAUGCCUUCCUGAUCGAUAGGAUUAACUGGAUCA
GCGCCGGCGUGUUCCUGGAUAGCAACCAGACCGCUGAGAAUCCUGUGUUUACAGUGUUUAAAGAUAAUGAGAUCCUG
UAUAGAGCUCAGCUGGCCUCUGAAGAUACAAAUGCCCAAAAGACUAUUACCAACUGCUUCCUGCUGAAAAACAAGAUU
UGGUGCAUCUCCCUGGUGGAGAUCUACGAUACCGGGGACAACGUGAUUAGACCUAAGCUGUUCGCCGUGAAAAUCCC
UGAGCAGUGCACUGAGUAUAGACCUCAGACUGAGGGCGUGAGUAAUCUGGUGGGGCUCCCCAACAAUAUCUGCCUGC
AGAAAACAAGCAACCAGAUUCUGAAACCAAAGCUGAUUAGCUAUACUCUGCCAGUAGUAGGGCAGUCUGGAACUUGUA
UUACAGAUCCUCUGUUGGCCAUGGAUGAGGGCUAUUUUGCUUAUAGCCAUCUGGAGAGAAUUGGCUCUUGUAGUAG
AGGCGUGAGUAAGCAGAGAAUCAUCGGAGUCGGAGAAGUGCUGGACCGCGGCGAUGAGGUCCCCUCACUGUUCAUGA
CAAACGUGUGGACCCCACCCAACCCCAAUACCGUGUAUCACUGUAGCGCUGUCUAUAACAACGAAUUUUACUACGUGCU
GUGCGCUGUGUCCACCGUGGGGGACCCUAUCCUGAAUUCUACUUACUGGAGCGGCUCCCUGAUGAUGACAAGGCUGG
CCGUGAAGCCAAAAUCUAACGGAGGCGGCUAUAAUCAGCACCAGCUGGCCCUCCGCUCCAUUGAGAAAGGCAGAUAUG
AUAAAGUCAUGCCAUAUGGCCCUAGCGGAAUUAAGCAGGGCGACACACUCUACUUCCCCGCCGUUGGCUUCCUGGUGA
GGACUGAGUUCAAAUAUAAUGAUAGCAAUUGUCCAAUCACAAAAUGUCAGUACUCUAAGCCUGAGAAUUGCAGACUG
AGCAUGGGAAUUAGACCUAAUAGCCAUUACAUCCUGAGAUCCGGACUCCUGAAGUACAAUCUGUCCGACGGCGAAAAC
CCUAAGGUCGUCUUUAUUGAAAUUAGCGACCAGAGGCUGAGCAUCGGAAGUCCAAGUAAGAUCUACGAUUCUCUGGG
ACAGCCCGUGUUUUACCAGGCUUCAUUCUCCUGGGAUACCAUGAUUAAGUUUGGCGACGUGCUGACCGUCAAUCCUC
UCGUGGUGAAUUGGAGGAAUAAUACAGUGAUUAGUAGACCAGGGCAGAGCCAGUGCCCCAGGUUUAACACCUGCCCC
GAGAUUUGUUGGGAAGGCGUGUAUAAUGACGCCUUUCUGAUUGAUAGAAUUAACUGGAUUUCAGCCGGAGUGUUCC
UCGACAGUAACCAGACCGCUGAGAACCCCGUGUUCACAGUGUUUAAAGAUAAUGAGAUCCUGUAUAGAGCUCAGCUGG
CUAGCGAGGACACUAACGCCCAAAAGACAAUUACUAAUUGUUUCCUGCUGAAGAAUAAGAUCUGGUGUAUUAGUCUU
GUGGAAAUCUAUGAUACCGGAGAUAAUGUGAUCAGGCCUAAGCUGUUCGCCGUGAAGAUCCCUGAACAGUGCACCUA
ACUCGAGCUGGUACUGCAUGCACGCAAUGCUAGCUGCCCCUUUCCCGUCCUGGGUACCCCGAGUCUCCCCCGACCUCGG
GUCCCAGGUAUGCUCCCACCUCCACCUGCCCCACUCACCACCUCUGCUAGUUCCAGACACCUCCCAAGCACGCAGCAAUG
CAGCUCAAAACGCUUAGCCUAGCCACACCCCCACGGGAAACAGCAGUGAUUAACCUUUAGCAAUAAACGAAAGUUUAAC
UAAGCUAUACUAACCCCAGGGUUGGUCAAUUUCGUGCCAGCCACACCCUGGAGCUAGC

Figure S2 The mRNA sequence of NV2 encoding 2Fs as the antigen.

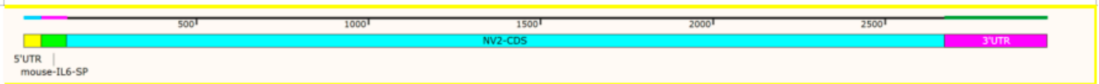

GAAUAAACUAGUAUUCUUCUGGUCCCCACAGACUCAGAGAGAACCCGCCACCAUGAAAUUUCUGAGCGCUAGGGACUU
UCACCCAGUGGCUUUCCUGGGCCUGAUGCUGGUGACCACCACCGCCAUUCUGCACUAUGAGAAACUGAGCAAGAUCGG
ACUGGUGAAGGGCGUGACACGGAAGUAUAAGAUUAAGUCUAAUCCCCUGACAAAGGACAUAGUGAUUAAGAUGAUCC
CUAAUGUGAGCAAUAUGAGCCAGUGCACCGGAUCUGUGAUGGAGAAUUACAAGACUAGACUGAACGGGAUCCUGACA
CCUAUCAAAGGCGCCCUGGAGAUCUACAAGAAUAACACCCAUGACCUCGUGGGCGACGUGAGACUGGCUGGCGUCAUC
AUGGCUGGGGUGGCAAUUGGCAUUGCCACUGCCGCCCAGAUCACAGCCGGCGUGGCUCUGUACGAAGCCAUGAAGAA
UGCCGACAACAUUAAUAAGCUGAAGUCUAGCAUUGAAUCUACAAACGAAGCCGUCGUGAAGCUCCAGGAAACUGCCGA
GAAAACCGUCUACGUGCUGACCGCUCUGCAGGACUAUAUCAAUACCAACCUGGUGCCAACCAUCGACAAAAUCUCCUGU
AAGCAGACAGAGCUGAGUCUGGAUCUCGCCCUCAGCAAGUAUCUGAGCGACCUGCUCUUCGUGUUCGGCCCCAACCUG
CAGGAUCCCGUGAGCAACAGCAUGACAAUCCAGGCCAUCUCCCAGGCUUUUGGCGGCAAUUACGAAACCCUGCUCAGG
ACAUUGGGCUACGCCACCGAGGAUUUCGAUGACCUGCUGGAGUCCGAUAGCAUCACUGGCCAGAUCAUCUACGUGGAU
CUGAGUAGCUACUACAUUAUUGUGCGCGUCUACUUCCCCAUCCUGACAGAGAUCCAGCAGGCCUAUAUCCAGGAGCUG
CUGCCAGUGAGCUUCAAUAAUGACAACUCUGAGUGGAUUUCUAUCGUGCCAAACUUUAUCCUGGUCCGGAAUACCCU
GAUCUCUAACAUUGAGAUUGGAUUUUGCCUGAUCACUAAGCGGAGUGUGAUUUGUAACCAGGAUUACGCUACACCUA
UGACCAACAACAUGAGAGAGUGUCUCACCGGCUCUACAGAAAAGUGUCCUAGAGAGCUGGUGGUUUCUAGUCACGUG
CCACGGUUUGCCCUGUCUAAUGGCGUUCUUUUCGCAAAUUGUAUUUCCGUGACCUGUCAGUGUCAGACCACCGGUCG
GGCCAUUUCCCAGAGCGGAGAGCAGACUCUUCUGAUGAUCGACAAUACUACAUGCCCAACAGCCGUGUUAGGCAACGU
GAUCAUCUCCUUGGGAAAAUAUCUGGGCUCUGUGAAUUAUAAUUCUGAGGGCAUCGCCAUUGGUCCCCCAGUGUUUA
UUCUCCACUACGAGAAGCUGAGUAAAAUCGGACUGGUGAAAGGCGUUACAAGAAAGUACAAGAUCAAGAGUAAUCCAC
UGACAAAAGAUAUCGUGAUCAAGAUGAUUCCAAACGUGAGCAACAUGAGCCAGUGUACAGGAUCUGUGAUGGAGAAC
UAUAAGACUCGGCUGAACGGUAUCCUGACACCCAUCAAAGGGGCUCUUGAAAUCUACAAAAACAAUACACACGAUUUG
GUUGGGGAUGUGAGACUGGCCGGAGUGAUCAUGGCUGGGGUGGCCAUCGGCAUUGCUACAGCUGCUCAGAUUACUG
CAGGCGUGGCCCUGUACGAAGCAAUGAAGAACGCUGAUAAUAUUAACAAGCUGAAGUCCUCUAUCGAGUCAACUAACG
AGGCCGUGGUUAAGCUACAGGAAACUGCCGAGAAAACUGUUUACGUGCUGACAGCCCUCCAAGAUUACAUCAAUACCA
AUCUGGUGCCAACCAUCGAUAAAAUCUCAUGCAAGCAGACCGAGCUGAGCCUGGAUCUCGCUCUGAGCAAAUACCUUA
GUGACCUGCUGUUCGUGUUCGGCCCCAACCUGCAGGAUCCUGUUUCAAAUAGCAUGACUAUUCAGGCCAUCUCUCAG
GCCUUCGGCGGCAACUACGAGACUCUGCUCAGAACCCUGGGCUACGCUACAGAGGACUUUGACGACUUGCUCGAAAGC
GACUCCAUUACCGGACAGAUCAUCUACGUGGACCUGAGCAGCUAUUAUAUUAUCGUGCGCGUGUACUUUCCCAUUCUG
ACUGAGAUUCAGCAGGCCUACAUUCAGGAGCUGCUGCCAGUGUCUUUCAAUAAUGAUAACUCCGAGUGGAUCUCCAU
CGUCCCCAAUUUCAUCCUGGUUAGAAAUACCCUGAUCAGUAACAUCGAGAUCGGCUUUUGUCUGAUUACAAAAAGGU
CUGUGAUCUGCAACCAGGAUUACGCCACUCCUAUGACCAACAACAUGCGCGAGUGUCUUACCGGAUCUACAGAGAAGU
GUCCUAGAGAACUCGUGGUGAGCAGCCAUGUGCCAAGAUUCGCUCUUUCCAACGGAGUGCUGUUUGCCAAUUGCAUC
UCCGUGACCUGCCAGUGUCAGACCACAGGAAGAGCAAUCUCUCAGAGUGGCGAACAGACCCUGCUGAUGAUCGAUAAC
ACAACCUGUCCCACAGCCGUGCUGGGCAAUGUGAUUAUCAGUCUGGGCAAGUACCUGGGCAGCGUGAAUUAUAAUUC
UGAGGGCAUCGCAAUCGGCCCUCCCGUGUUUAACUCGAGCUGGUACUGCAUGCACGCAAUGCUAGCUGCCCCUUUCC
CGUCCUGGGUACCCCGAGUCUCCCCCGACCUCGGGUCCCAGGUAUGCUCCCACCUCCACCUGCCCCACUCACCACCUCUG
CUAGUUCCAGACACCUCCCAAGCACGCAGCAAUGCAGCUCAAAACGCUUAGCCUAGCCACACCCCCACGGGAAACAGCAG
UGAUUAACCUUUAGCAAUAAACGAAAGUUUAACUAAGCUAUACUAACCCCAGGGUUGGUCAAUUUCGUGCCAGCCACA
CCCUGGAGCUAGC

Figure S3 B-cell Linear Epitopes and MHC Epitopes of NiV G/F Analyzed Using the IEDB Database.

B cell

G Protein	Start	End	Sequence		F Protein	Start	End	Sequence
1	1	3	MPA		1	152	153	ES
2	13	20	TTSDKGKI		2	218	223	LQDPVS
3	75	78	RSTD		3	251	251	E
4	171	176	YRPQTE		4	302	304	NND
5	271	278	WTPPNPNT		5	345	347	ATP
6	324	330	KSNGGGY		6	361	362	EK
7	350	351	PY		7	400	404	AISQS
8	353	357	PSGIK		8	438	438	Y
9	390	390	S		9	443	444	IA
10	420	423	DGEN		10	449	450	VF
11	443	443	K		11	468	468	Q
12	488	492	PGQSQ		12	533	538	VRPTSS
13	530	533	QTAE					
14	554	558	EDTNA					

MHC

G Protein	Start	End	Allele	Median binding percentile	Peptide	MHC
1	103	111	HLA-B*40:01	0.01	TEIGPKVSL	MHCI
2	532	540	HLA-B*44:03	0.01	AENPVFTVF	MHCI
3	471	479	HLA-B*57:01	0.01	TVNPLVVNW	MHCI
4	20	28	HLA-B*35:01	0.01	IPSKVIKSY	MHCI
5	532	540	HLA-B*44:02	0.01	AENPVFTVF	MHCI
6	471	479	HLA-B*58:01	0.01	TVNPLVVNW	MHCI
7	199	207	HLA-B*07:02	0.01	KPKLISYTL	MHCI
8	197	205	HLA-B*15:01	0.01	ILKPKLISY	MHCI
9	558	566	HLA-B*15:01	0.01	AQKTITNCF	MHCI
10	134	142	HLA-A*68:02	0.01	STASINENV	MHCI
11	302	310	HLA-B*53:01	0.01	DPILNSTYW	MHCI
12	284	292	HLA-A*30:02	0.01	AVYNNEFYY	MHCI
13	471	479	HLA-A*32:01	0.01	TVNPLVVNW	MHCI
14	532	540	HLA-B*40:01	0.02	AENPVFTVF	MHCI
15	512	520	HLA-A*02:03	0.02	FLIDRINWI	MHCI
16	223	231	HLA-A*01:01	0.02	AMDEGYFAY	MHCI
17	435	443	HLA-A*03:01	0.02	RLSIGSPSK	MHCI
18	408	416	HLA-B*15:01	0.02	ILRSGLLKY	MHCI
19	452	460	HLA-A*23:01	0.02	VFYQASFSW	MHCI
20	423	431	HLA-B*51:01	0.02	NPKVVFIEI	MHCI
21	226	240	HLA-DPA1*02:01/DPB1*01:01	0.02	EGYFAYSHLERIGSC	MHCII
F Protein	Start	End	Allele	Median binding percentile	Peptide	MHC
1	163	171	HLA-A*68:02	0.01	ETAEKTVYV	MHCI
2	280	288	HLA-A*24:02	0.01	VYFPILTEI	MHCI
3	280	288	HLA-A*23:01	0.01	VYFPILTEI	MHCI
4	47	55	HLA-A*03:01	0.01	KIKSNPLTK	MHCI
5	124	132	HLA-A*68:02	0.01	TAAQITAGV	MHCI
6	47	55	HLA-A*30:01	0.01	KIKSNPLTK	MHCI
7	480	488	HLA-A*02:01	0.02	LLDTVNPSL	MHCI
8	32	40	HLA-A*03:01	0.02	KLSKIGLVK	MHCI
9	133	141	HLA-A*02:03	0.02	ALYEAMKNA	MHCI
10	198	206	HLA-A*01:01	0.02	SLDLALSKY	MHCI
11	284	292	HLA-B*15:01	0.02	ILTEIQQAY	MHCI
12	162	170	HLA-B*44:02	0.02	QETAEKTVY	MHCI
13	91	105	HLA-DRB1*15:01	0.04	KGALEIYKNNTHDLV	MHCII
14	306	320	HLA-DRB1*15:01	0.09	SEWISIVPNFILVRN	MHCII

Figure S4 Docking prediction of the NiV G/F protein in complex with the corresponding antibody, performed using AlphaFold3.

A: NiV-F and anti-NiV-F antibody

B: NiV-G and anti-NiV-G antibody

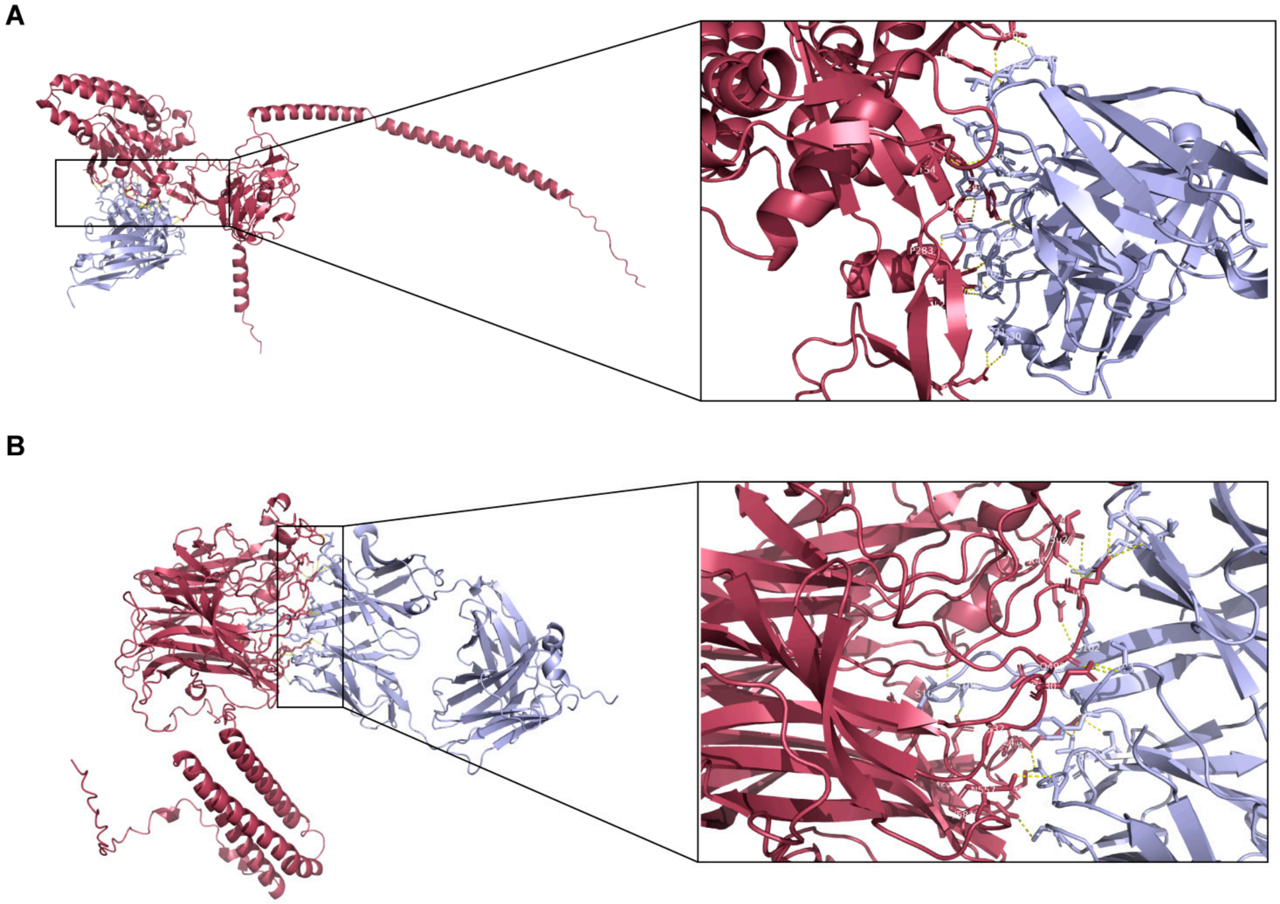

Figure S5 Characterization of NV1 mRNA-LNPs

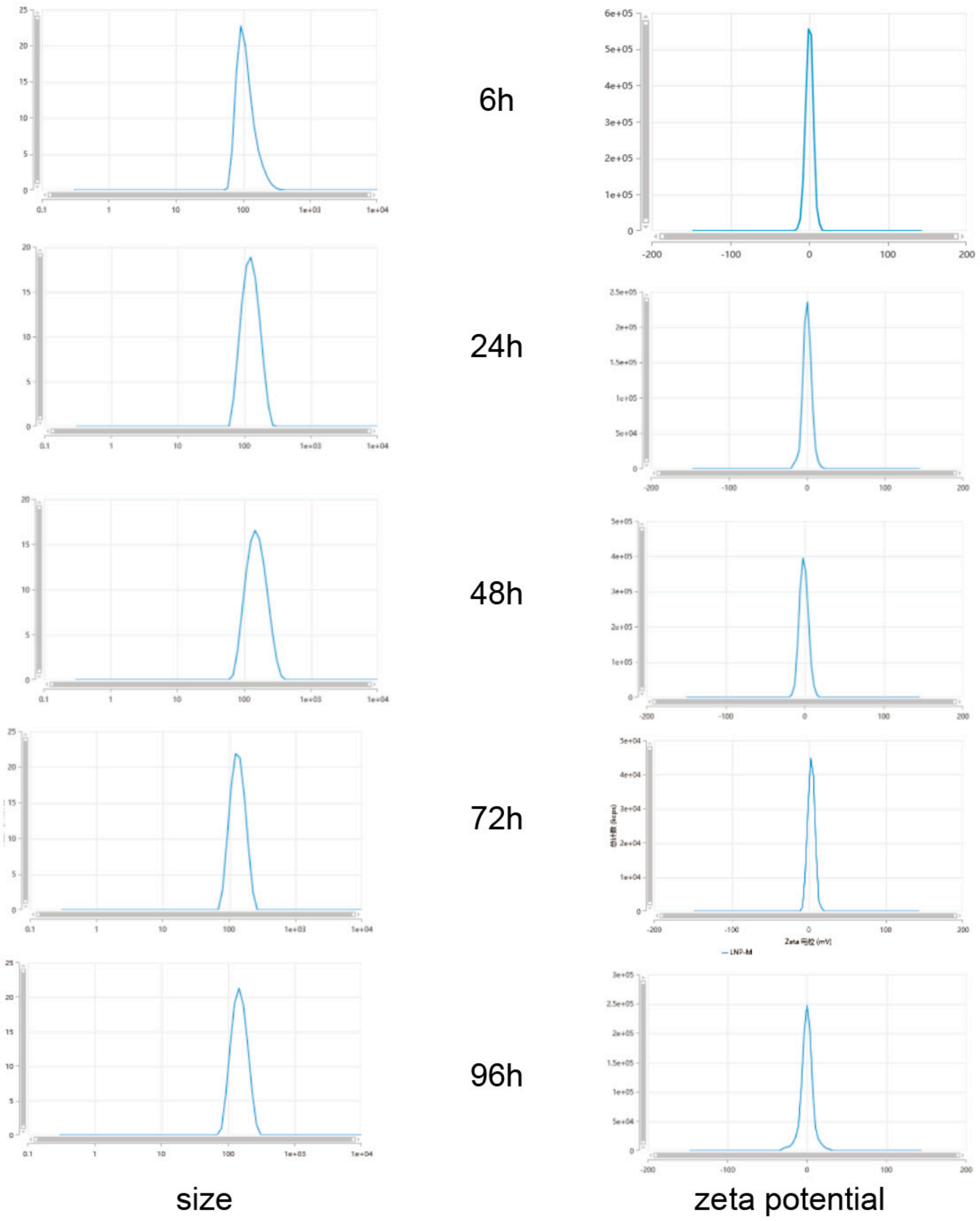

Encapsulation rate（%）

	6h	24h	48h	72h	96h
NV1	98.3	98.8	98.9	96.5	93.5
	98.5	98.4	98.5	96.3	94.7
	97.2	96.6	98.1	96.2	94.2

Figure S6 Characterization of NV2 mRNA-LNPs

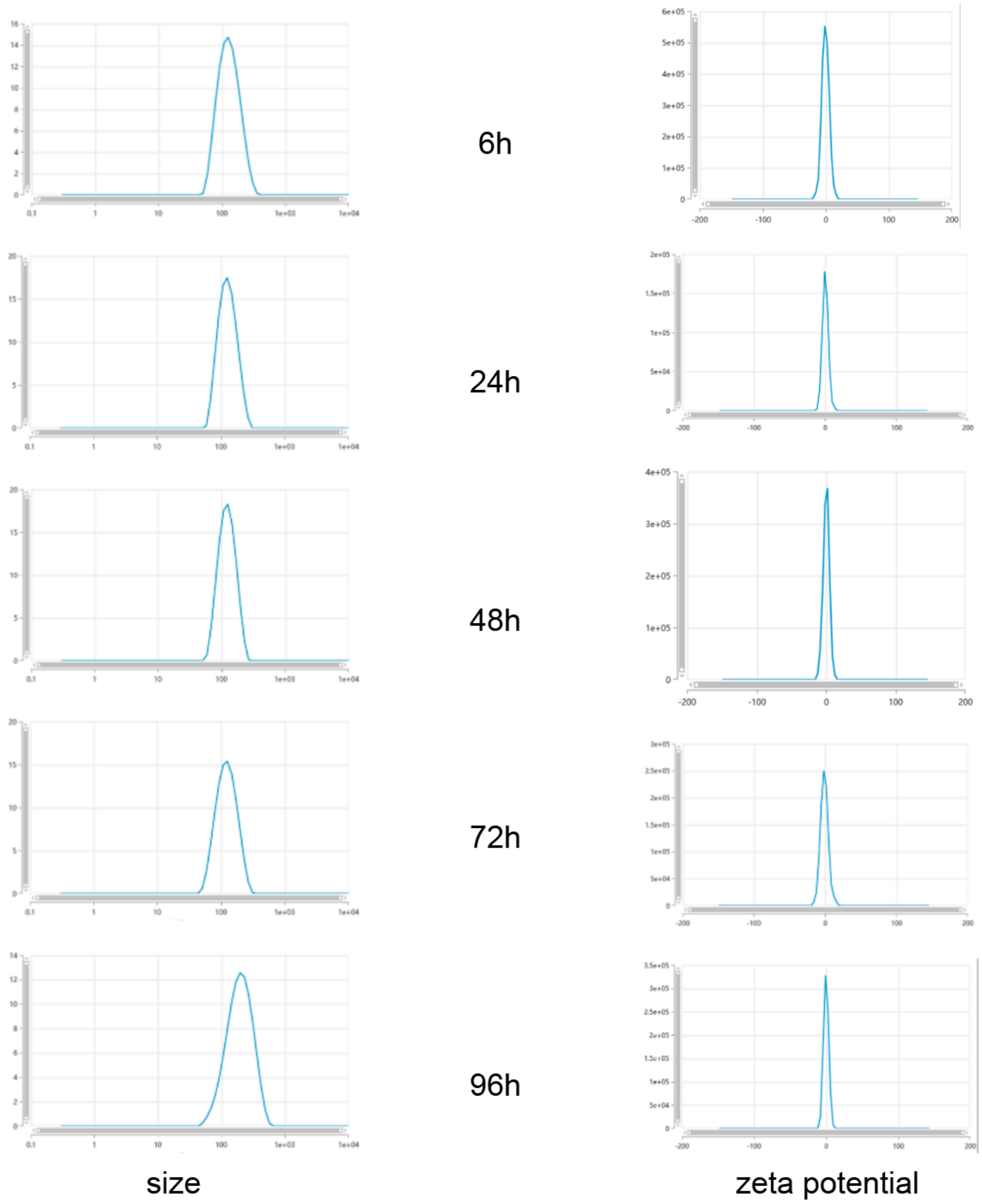

Encapsulation rate（%）

	97.3	98.6	97.3	97.4	94.6
NV2	97.5	97.5	97.4	97.1	94.1
	98.7	95.2	94.9	94.3	92.9

Figure S7 Full-length Western blot membranes showing the demonstration of protein expression in vitro.

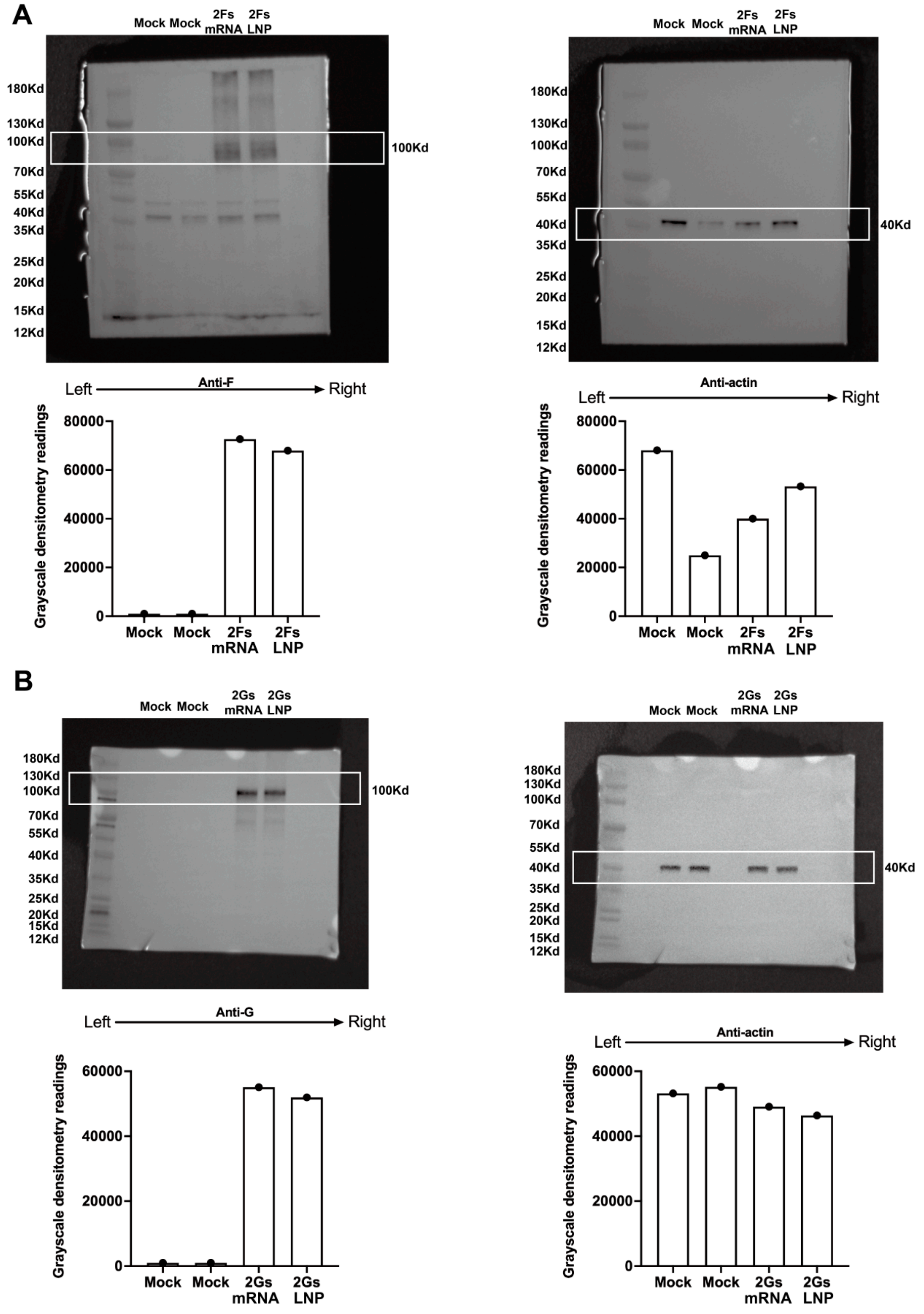

A. Whole-membrane WB images and densitometry analysis for Mock and 2Fs.

B. Whole-membrane WB images and densitometry analysis for Mock and 2Gs.

FigureS8 Heatmap showing the dynamic changes in serum cytokine levels at 5 h and 24h post-initial immunization (n=5). Data are log2-transformed; log2FC (fold change) represents the ratio of cytokine levels at 5h relative to 24h post-initial immunization.

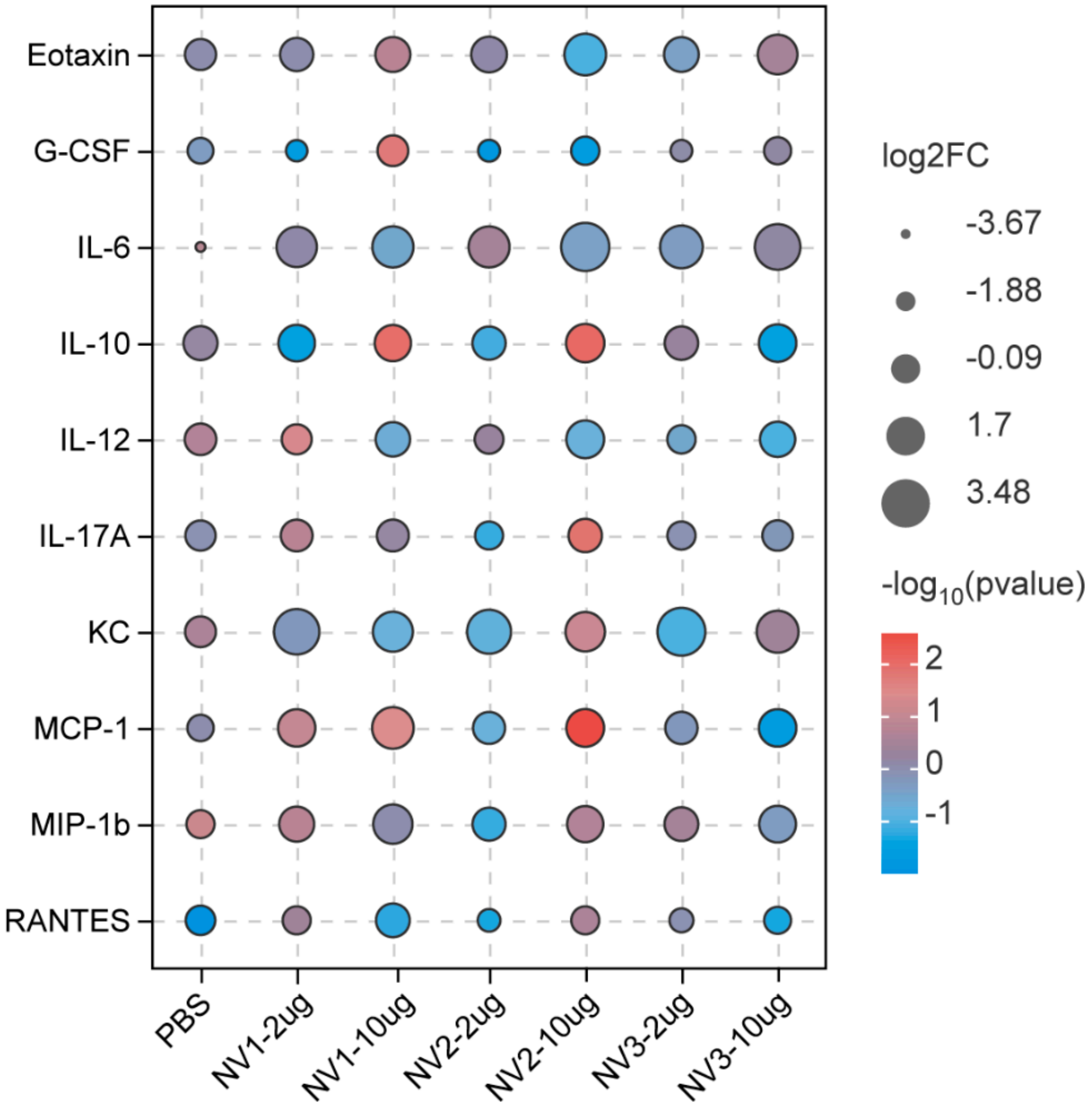

Figure S9 Pairwise statistical comparisons of serum cytokine levels across experimental groups at 5 h and 24 h post-initial immunization.

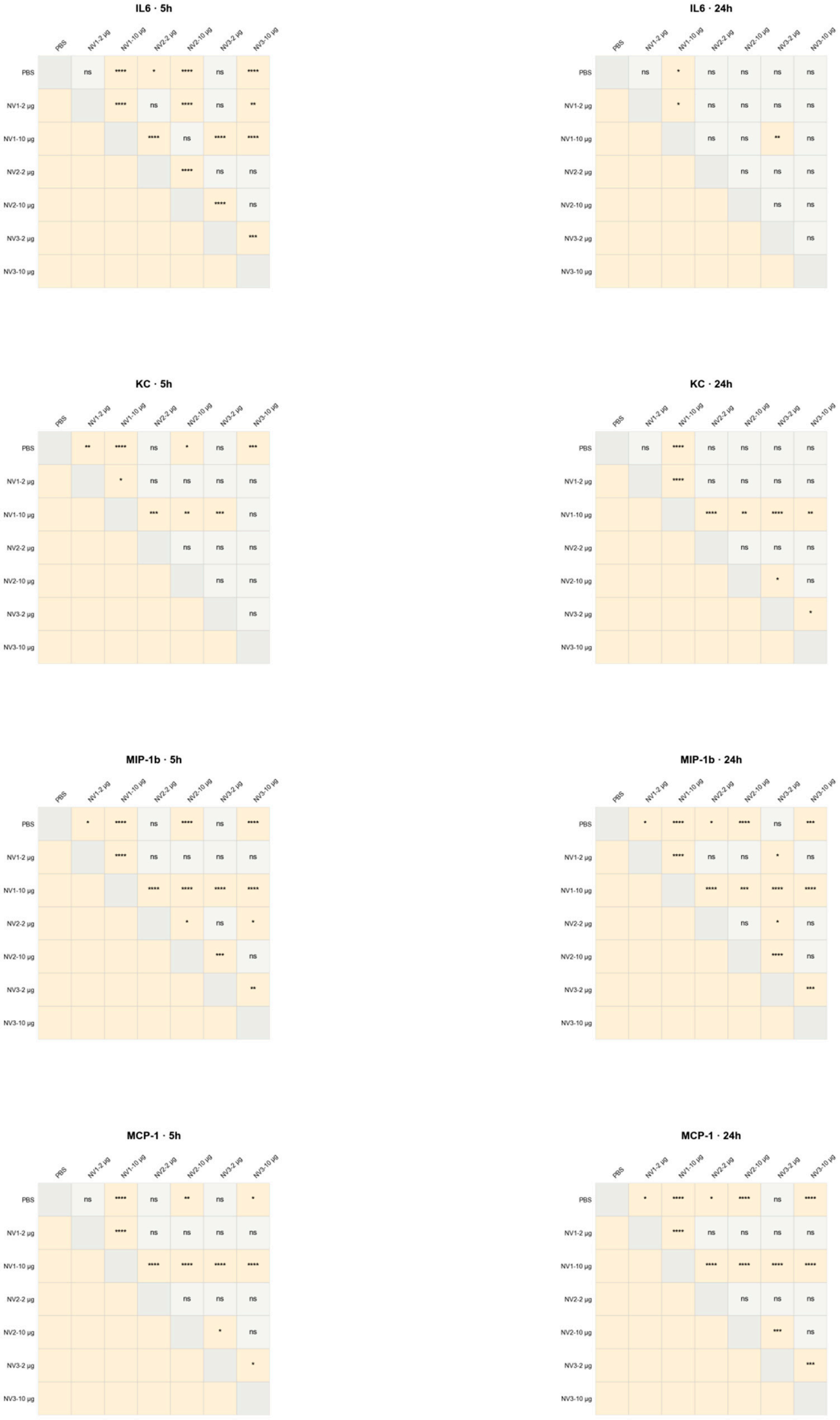

Figure S10 Representative IFN-γ ELISpot images from the cellular immunogenicity assessment.

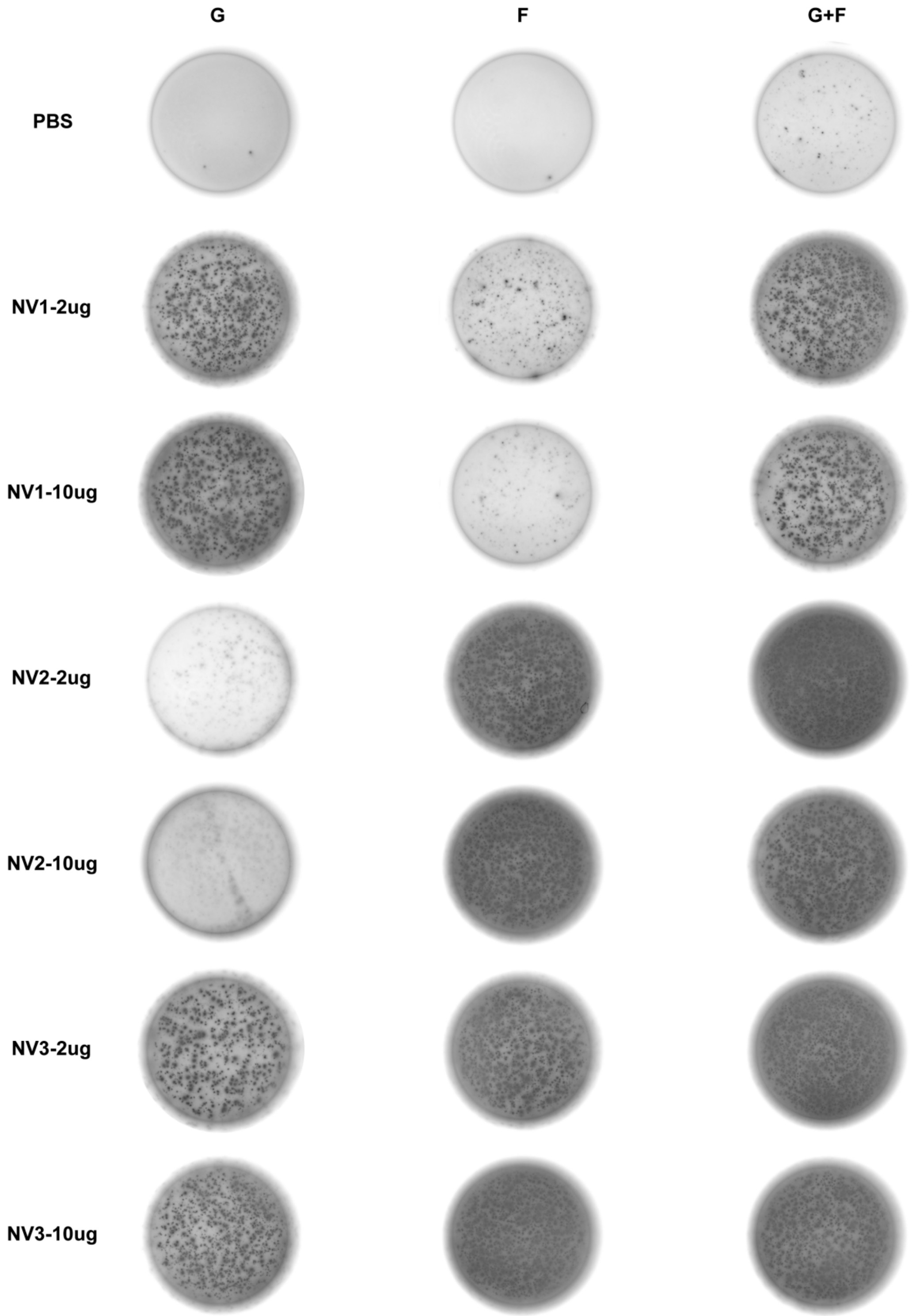

Figure S11 Representative IL-2 ELISpot images from the cellular immunogenicity assessment.

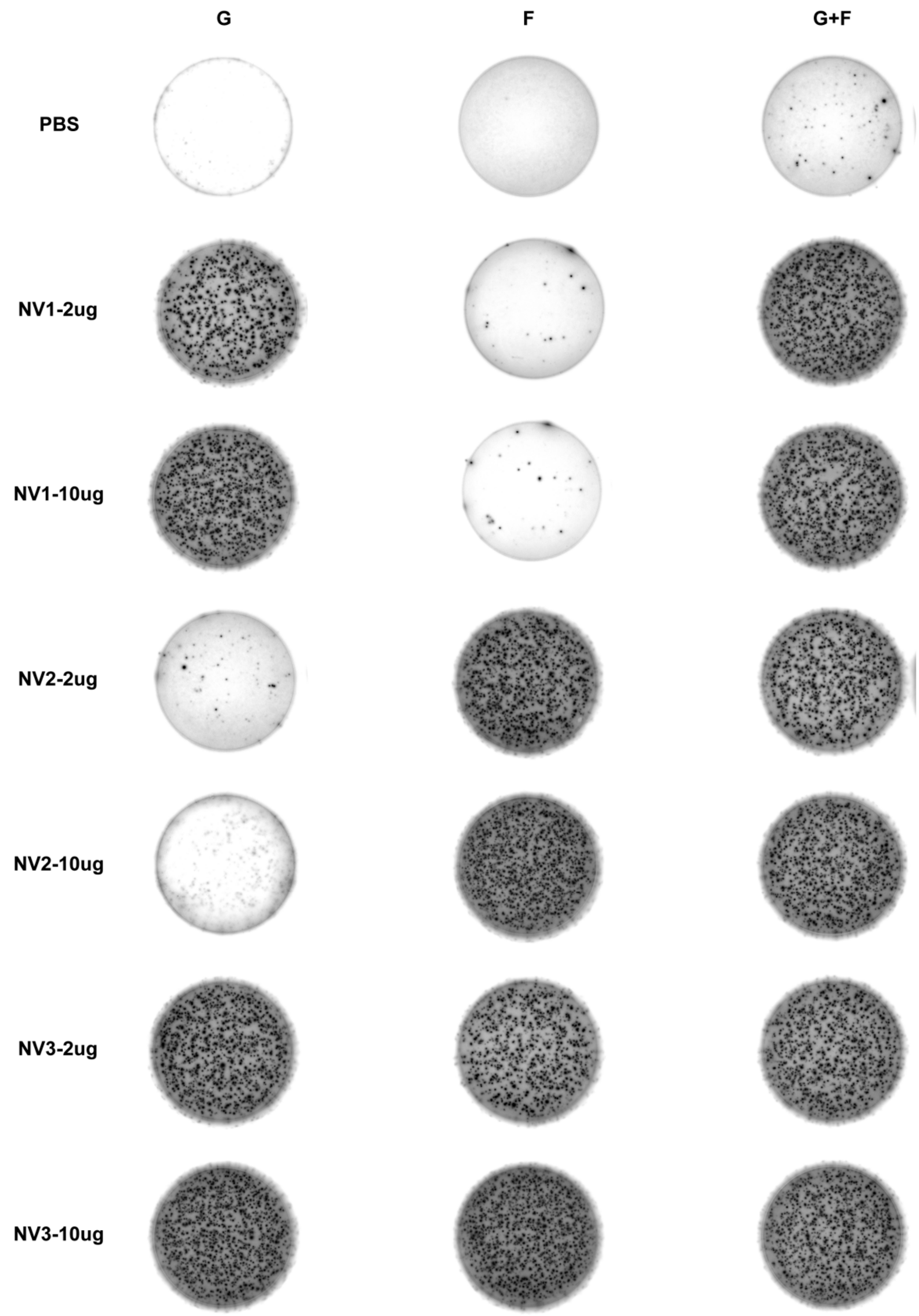

Figure S12 Representative IL-4 ELISpot images from the cellular immunogenicity assessment.

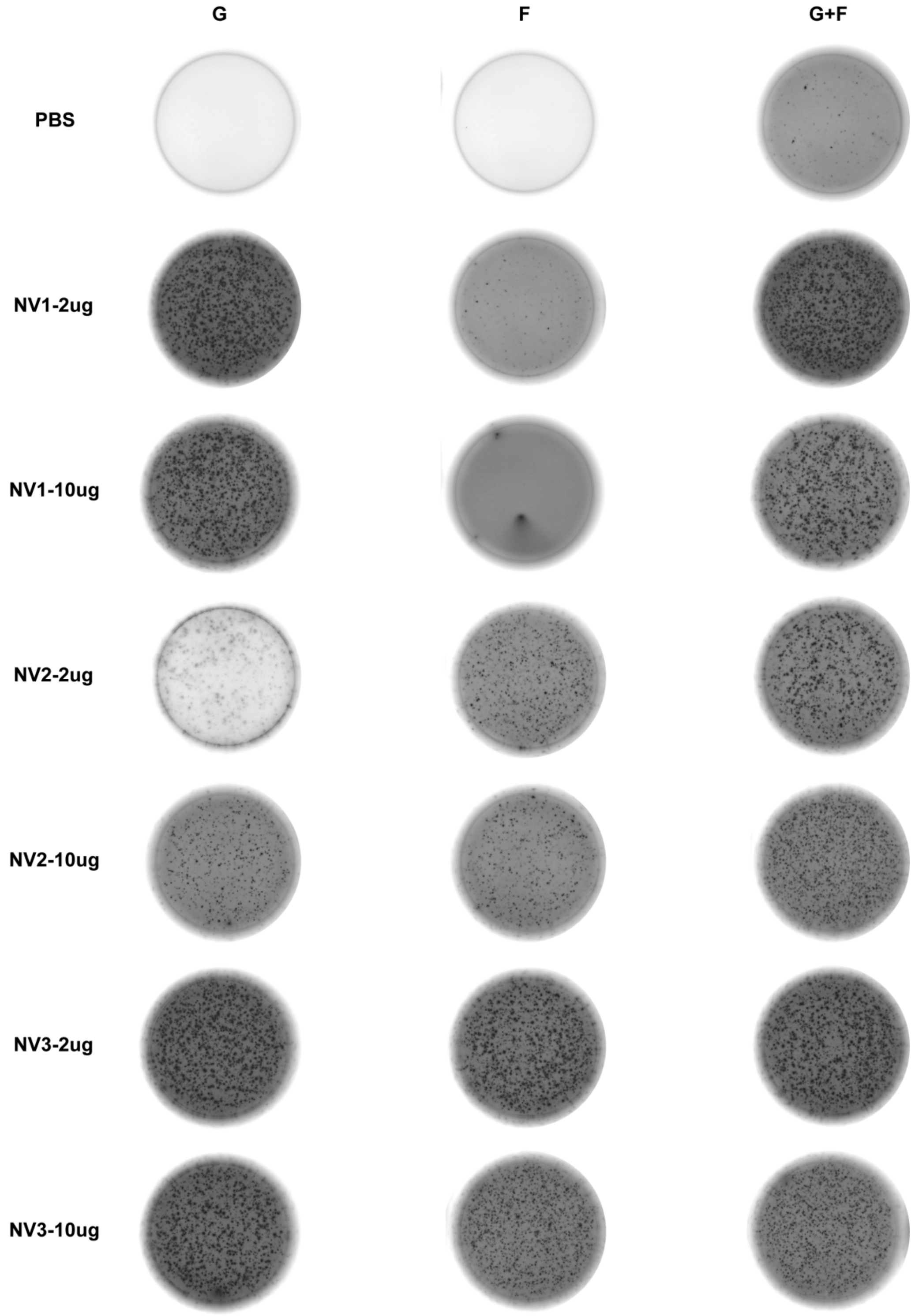

Figure S13 Representative ELISpot images and spot counts using PHA stimulation as a positive control.

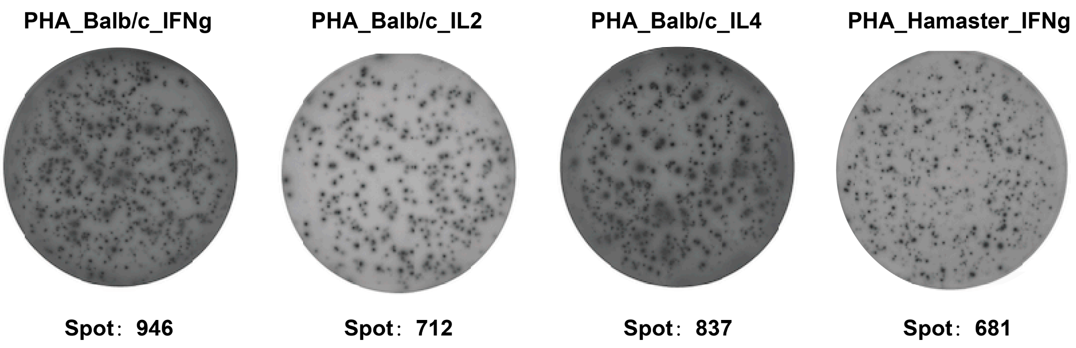

Figure S14 Statistical significance analysis of ELISpot assays using different stimuli in immunized BALB/c mice.

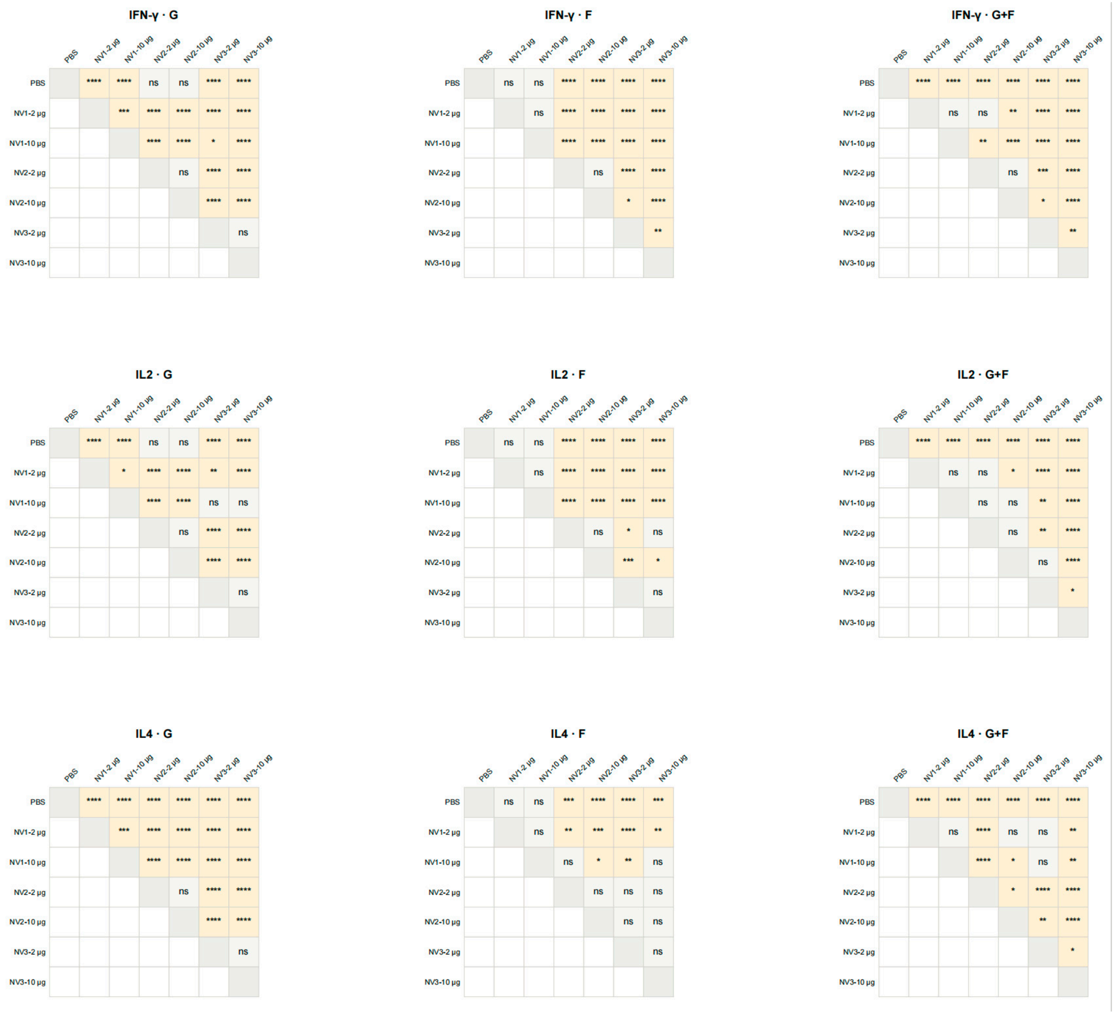

Figure S15 Statistical analysis of antibody isotype (IgG/IgM/IgA) proportions within IGHV gene families in BCR-seq data. Bars indicate mean ± SEM; significance was assessed by one-way ANOVA with Tukey's post hoc test.）

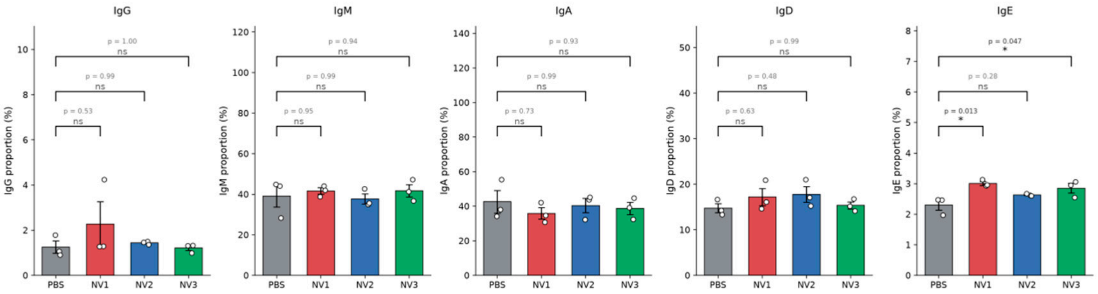

Figure S16 CDR3 length distribution and conserved CDR3 sequences across different immunization groups.

Control

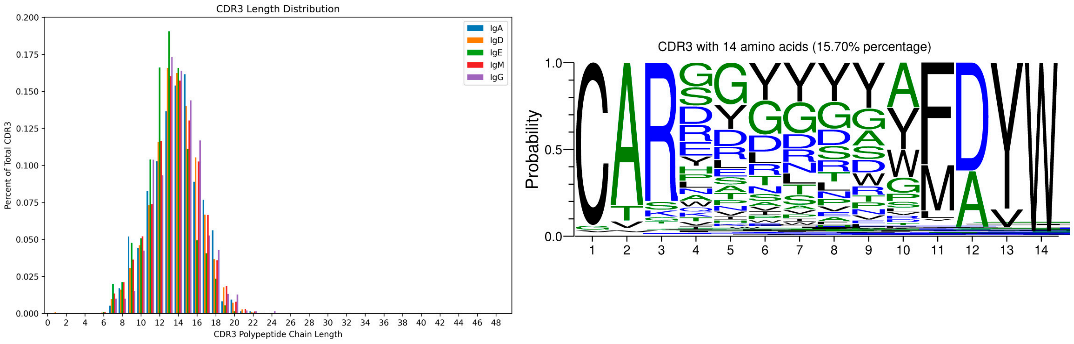

NV1

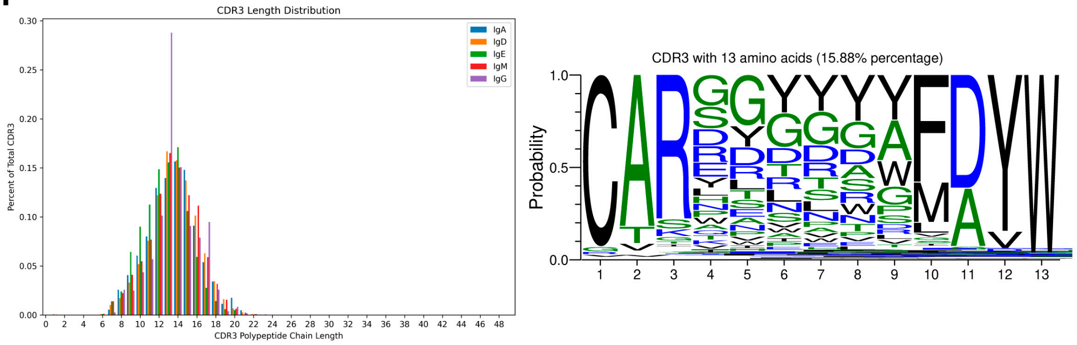

NV2

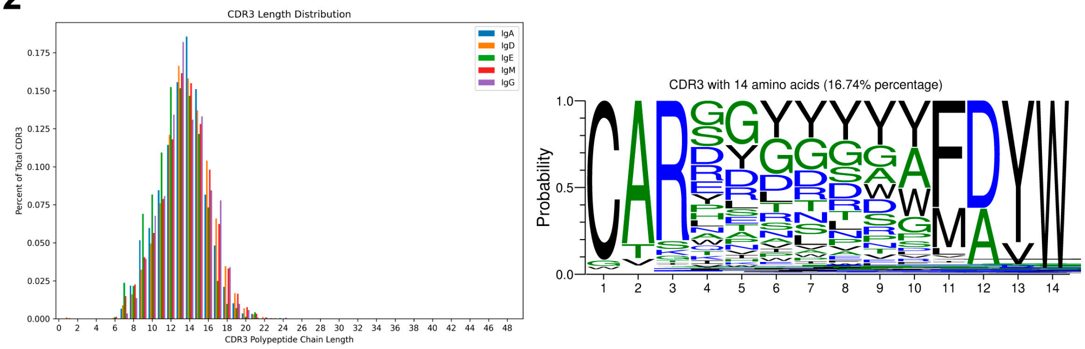

NV3

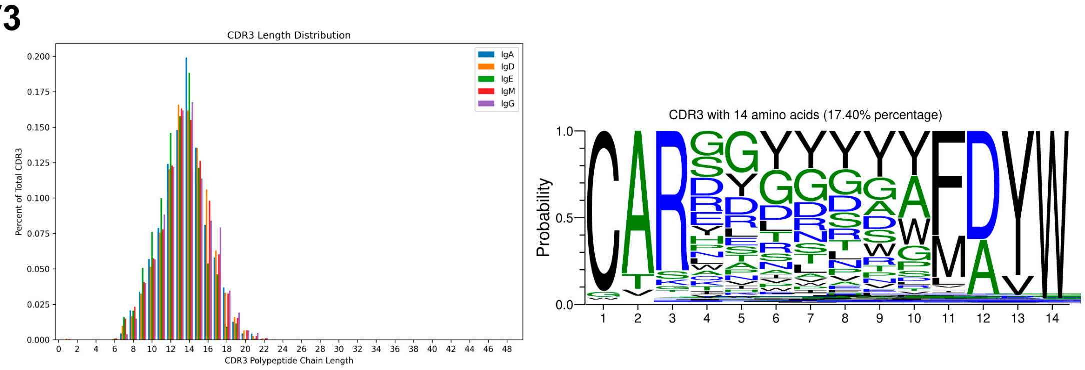

Figure S17 Top 10 V-J gene pairing frequencies in IgM and IgD repertoires across different groups.

A. Top 10 V-J gene pairing frequencies in IgD repertoires across different groups.

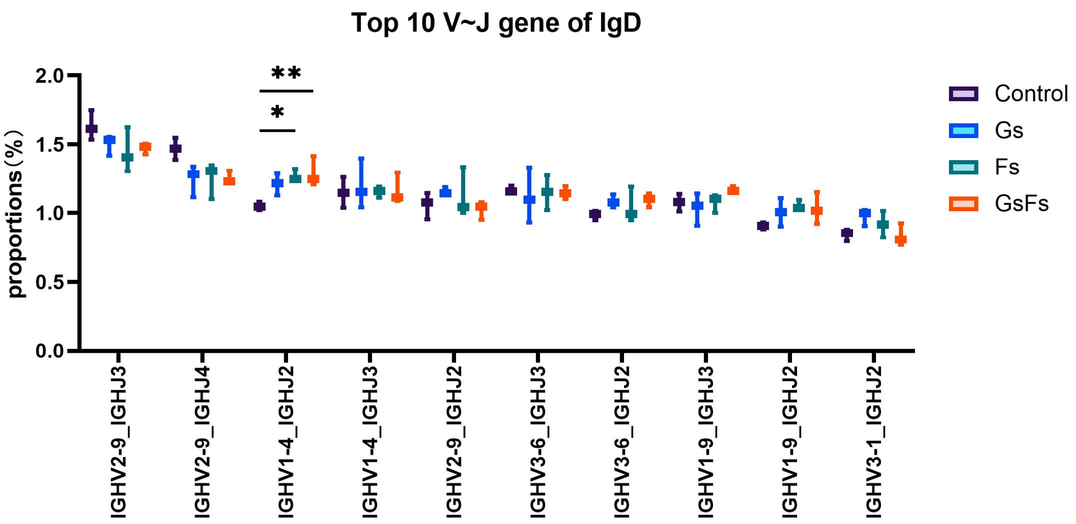

B. Top 10 V-J gene pairing frequencies in IgM repertoires across different groups.

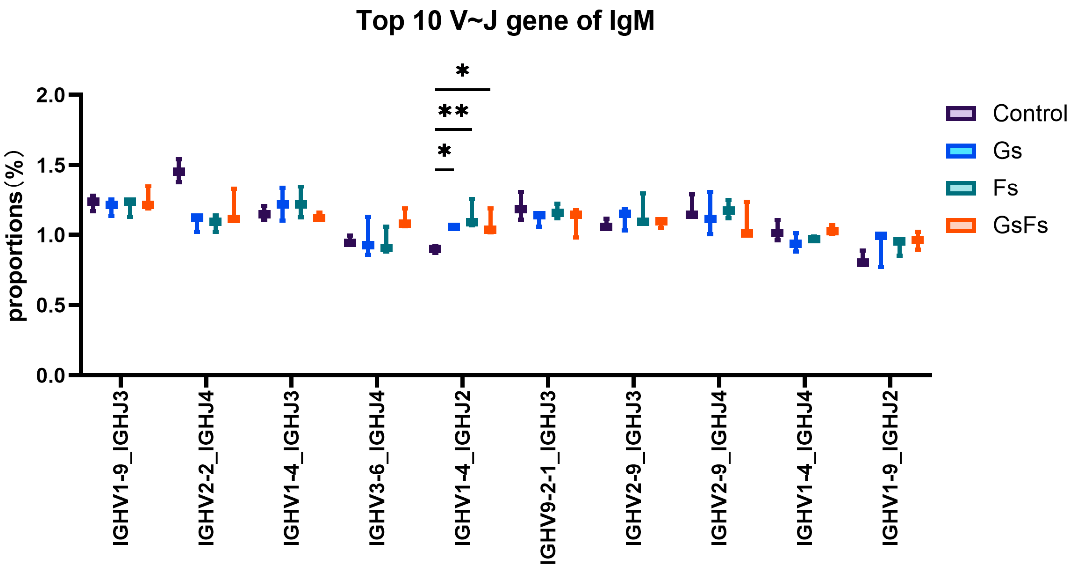

Figure S18 Body temperature changes of BALB/c mice on days 1–5 post initial immunization

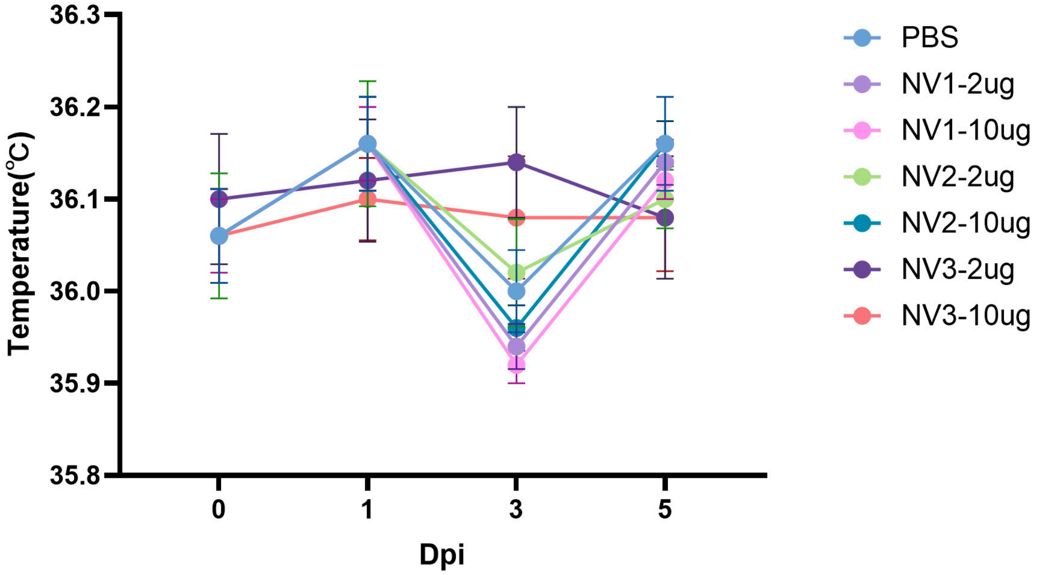

Figure S19 Body weight changes of BALB/c mice on days 1–5 post initial immunization

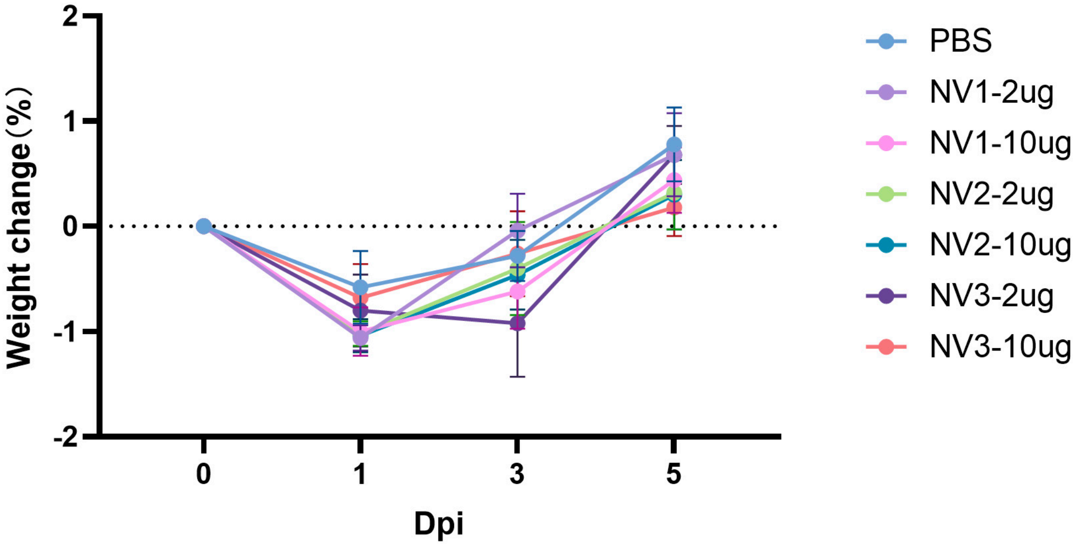

Figure S20 Statistical significance analysis among groups in the hamster ELISpot assay

IFN-γ · G

	PBS	NV1-2 µg	NV1-10 µg	NV2-2 µg	NV2-10 µg	NV3-2 µg	NV3-10 µg
PBS		****	****	ns	ns	****	****
NV1-2 µg			ns	****	****	ns	****
NV1-10 µg				****	****	ns	**
NV2-2 µg					ns	****	****
NV2-10 µg						****	****
NV3-2 µg							**
NV3-10 µg							

IFN-γ · F

	PBS	NV1-2 µg	NV1-10 µg	NV2-2 µg	NV2-10 µg	NV3-2 µg	NV3-10 µg
PBS		ns	ns	****	****	****	****
NV1-2 µg			ns	****	****	****	****
NV1-10 µg				****	****	****	****
NV2-2 µg					ns	ns	ns
NV2-10 µg						ns	ns
NV3-2 µg							ns
NV3-10 µg							

IFN-γ · F+G

	PBS	NV1-2 µg	NV1-10 µg	NV2-2 µg	NV2-10 µg	NV3-2 µg	NV3-10 µg
PBS		***	***	****	****	****	****
NV1-2 µg			ns	*	ns	*	*
NV1-10 µg				ns	ns	ns	ns
NV2-2 µg					ns	ns	ns
NV2-10 µg						ns	ns
NV3-2 µg							ns
NV3-10 µg							
